# The transmembrane protein LRIG1 triggers melanocytic tumor development following chemically induced skin carcinogenesis

**DOI:** 10.1002/1878-0261.12945

**Published:** 2021-03-31

**Authors:** Christine Hoesl, Thomas Fröhlich, Christian Posch, Hermann Kneitz, Matthias Goebeler, Marlon R. Schneider, Maik Dahlhoff

**Affiliations:** ^1^ Institute of Molecular Animal Breeding and Biotechnology Gene Center LMU München Germany; ^2^ Laboratory for Functional Genome Analysis (LAFUGA) Gene Center LMU München Germany; ^3^ Klinik und Poliklinik für Dermatologie und Allergologie Klinikum rechts der Isar – TU München Germany; ^4^ Faculty of Medicine Sigmund Freud Universität Wien Austria; ^5^ Klinik und Poliklinik für Dermatologie, Venerologie und Allergologie Universitätsklinikum Würzburg Germany; ^6^ Institute of In vivo and In vitro Models University of Veterinary Medicine Vienna Austria

**Keywords:** ERBB receptors, LRIG1, melanoma, mouse model, skin carcinogenesis

## Abstract

The incidence of melanoma and nonmelanoma skin cancer has increased tremendously in recent years. Although novel treatment options have significantly improved patient outcomes, the prognosis for most patients with an advanced disease remains dismal. It is, thus, imperative to understand the molecular mechanisms involved in skin carcinogenesis in order to develop new targeted treatment strategies. Receptor tyrosine kinases (RTK) like the ERBB receptor family, including EGFR/ERBB1, ERBB2/NEU, ERBB3, and ERBB4, are important regulators of skin homeostasis and their dysregulation often results in cancer, which makes them attractive therapeutic targets. Members of the leucine‐rich repeats and immunoglobulin‐like domains protein family (LRIG1‐3) are ERBB regulators and thus potential therapeutic targets to manipulate ERBB receptors. Here, we analyzed the function of LRIG1 during chemically induced skin carcinogenesis in transgenic mice expressing LRIG1 in the skin under the control of the keratin 5 promoter (LRIG1‐TG mice). We observed a significant induction of melanocytic tumor formation in LRIG1‐TG mice and no difference in papilloma incidence between LRIG1‐TG and control mice. Our findings also revealed that LRIG1 affects ERBB signaling via decreased phosphorylation of EGFR and increased activation of the oncoprotein ERBB2 during skin carcinogenesis. The epidermal proliferation rate was significantly decreased during epidermal tumorigenesis under LRIG1 overexpression, and the apoptosis marker cleaved caspase 3 was significantly activated in the epidermis of transgenic LRIG1 mice. Additionally, we detected LRIG1 expression in human cutaneous squamous cell carcinoma and melanoma samples. Therefore, we depleted LRIG1 in human melanoma cells (A375) by CRISPR/Cas9 technology and found that this caused EGFR and ERBB3 downregulation in A375 LRIG1 knockout cells 6 h following stimulation with EGF. In conclusion, our study demonstrated that LRIG1‐TG mice develop melanocytic skin tumors during chemical skin carcinogenesis and a deletion of LRIG1 in human melanoma cells reduces EGFR and ERBB3 expression after EGF stimulation.

## Graphical abstract

The tyrosine kinase inhibitor *leucine‐rich repeats and immunoglobulin‐like domains 1* (LRIG1) were overexpressed in the mouse skin to assess its function in this organ. While LRIG1 efficiently inactivated EGFR, epidermal LRIG1 excess showed no impact in experimentally induced skin carcinogenesis. However, transgenic animals developed remarkable melanocytic nevi induced by cleaved LRIG1, indicating a potential role for LRIG1 in melanoma.

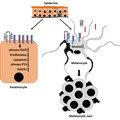

**Abbreviations**
AKT: RAC‐alpha serine/threonine‐protein kinase
CASP3: caspase 3
cSCC: cutaneous squamous cell carcinoma
DAPI: 4′,6‐diamidino‐2‐phenylindole
DMBA: 7,12‐dimethylbenz(a)anthracene
EGF: epidermal growth factor
EGFR: EGF receptor
FCS: fetal calf serum
GAPDH: glyceraldehyde‐3‐phosphate dehydrogenase
H&E: hematoxylin and eosin
HF: hair follicle
IFE: interfollicular epidermis
KO: knockout
KRT: keratin
LOR: loricrin
LRIG: leucine‐rich repeats and immunoglobulin‐like domains
MAPK1/2: mitogen‐activated protein kinase 1/2
MKI67: proliferation marker protein Ki‐67
MLANA: Melan‐A
NMSC: nonmelanoma skin cancer
NOTCH1: neurogenic locus notch homolog protein 1
PCNA: proliferating cell nuclear antigen
PTEN: phosphatidylinositol 3,4,5‐triphosphate 3‐phosphatase and dual specificity protein phosphatase PTEN
RTK: receptor tyrosine kinase
TG: transgenic
TP53: cellular tumor antigen p53
TPA: 12‐*O*‐tetra‐decanoylphorbol‐13‐acetate

## 1 Introduction

Melanoma and cutaneous squamous cell carcinoma (cSCC) of the skin are the most prominent types of skin cancer, with dramatically increasing incidences in recent years [1]. Worldwide, there are about 132 000 newly diagnosed melanoma cases per year and 2–3 million new incidences of nonmelanoma skin cancer (NMSC). Today, there are several therapeutic strategies like immune checkpoint and kinase inhibitors to improve patients' outcome. However, prognosis is still very poor for advanced tumors. It is thus indispensable to understand the molecular mechanisms involved in skin carcinogenesis to develop new targeted treatment strategies.

Receptor tyrosine kinases (RTKs) like the epidermal growth factor receptor (EGFR) and its family members ERBB2‐4 (HER2‐4) are crucial players during NMSC [2, 3] as well as melanoma development [4]. The ERBB receptor family is widely expressed in skin [5] and involved in pivotal processes like proliferation, differentiation, and cell death to maintain epidermal homeostasis [6]. Also, the RTKs of the ERBB family are often dysregulated in tumors [7] and therefore prominent targets in cancer therapy [8]. EGFR affects epidermal tumor development and progression [9] and *EGFR* expression is increased in cSCC [10]. Similarly, the skin‐specific deletion of ERBB2 and ERBB3 influenced skin cancer promotion [2, 3]. Importantly, feedback mechanisms of the complex ERBB signaling network are frequently affected during tumorigenesis [11]. We investigated the role of leucine‐rich repeats and immunoglobulin‐like domains protein 1 (LRIG1) regulating ERBB network.

LRIG1 is one of three members of a transmembrane protein family (LRIG1‐3) [12] and a negative regulator of ERBB receptors [13, 14]. Additionally, LRIG1 promotes stem cell quiescence in the intestine [15], the stomach [16], and also in the skin [17]. *Lrig1* knockout mice show a severe epidermal, psoriasis‐like phenotype [18]. In previous studies, we have shown that the skin‐specific overexpression of LRIG1 affects skin morphogenesis as well as homeostasis by regulating ERBB signaling [13]. Moreover, LRIG1 upregulation in consequence of the skin‐specific overexpression of the EGFR ligand epigen during homeostasis [19], or owing to increased intrinsic EGFR signaling in epidermal papillomas of *Egfr^Dsk5^* mice [9], indicates a function of LRIG1 in feedback regulation of the ERBB network also in the skin. Furthermore, LRIG proteins arouse attention due to their potential as prognostic factors in various tumors [20]. LRIG1 is thought to be a tumor suppressor in non‐small‐cell lung cancer [21], cervical SCC [22], and also melanoma [23] and cSCC [24]. While metastasis and decreased survival are shown in cSCC patients with low LRIG1 expression, the upregulation of LRIG1 is related to better prognosis and more differentiated tumors [24]. The prognostic value of LRIGs is often also associated with ERBB signaling like in urinary bladder [25] or breast cancer [26]. In glioma, loss of LRIG1 results in very aggressive tumors [27], though the ectopic LRIG1 expression causes decreased tumor cell proliferation by negatively regulating the oncogenic EGFR mutant EGFRvIII, which is often highly expressed in glioblastoma [28].

Taken together, while LRIG1 might be a promising target for tumor therapy in the skin by influencing the ERBB signaling network, the molecular function and the underlying mechanisms are largely unknown. Therefore, we investigated the effect of LRIG1 overexpression in mouse skin during epidermal carcinogenesis in a 7,12‐dimethylbenz(a)anthracene/12‐*O*‐tetra‐decanoylphorbol‐13‐acetate (DMBA/TPA) chemical carcinogenesis model. Our data indicate a tumorigenic function of LRIG1 in the skin.

## 2 Materials and methods

### 2.1 Cells

Human HaCaT keratinocytes, A431 squamous cell carcinoma cells, and A375 melanoma cells were purchased from CLS (Cell Lines Service, Eppelheim, Germany). Short tandem repeat DNA profiling analysis was used to authenticate all human permanent cell lines in the CLS cell bank. The human melanoma cell lines SK‐MEL2 and SK‐MEL28 were kindly provided by the Department of Dermatology and Allergy, Technical University of Munich, Germany. Mycoplasma testing with a mycoplasma detection kit (PlasmoTest, InvivoGen, Toulouse, France) was regularly performed on all cultured cells, and all used cell lines were found to be mycoplasma free. All cells were cultured in DMEM^®^ medium (Biochrom, Berlin, Germany) supplemented with 10% fetal calf serum (FCS; Biochrom), penicillin (100 U·mL^−1^), and streptomycin (100 µg·mL^−1^) (Biochrom) in a humidified incubator with 5% CO_2_ at 37 ˚C.

### 2.2 Human samples

The study methodologies conformed to the standards set by the Declaration of Helsinki. Analysis of human cSCC tissue samples was approved by the Ethics Committee of the Medical Faculty, University of Würzburg, Germany (reference number #169/12). Melanoma biopsies were kindly provided by the Department of Dermatology and Allergy, Technical University of Munich, Germany, and obtained with the approval of the local Ethics Committee (307/18S).

The Department of Dermatology, University Hospital Würzburg, Germany, generously provided nondiseased skin samples of 10 individuals as controls and 10 cSCC samples of 10 patients between 71 and 92 years of age. Eight of these patients were diagnosed at stage I (pT1G1: 6 patients, pT1G2: 2 patients) and two at stage II (pT2G2 and pT2G3: one patient each) as classified according to the 8th Edition of the staging manual of the American Joint Committee on Cancer (AJCC‐8) [29]. Tissue was taken from the following anatomical sites: cheeks (three patients), forehead (three patients), and nose, ear, dorsum of the hand and lower leg (one patient each). Skin samples from nondiseased skin of 10 individuals served as controls.

### 2.3 Mice

C57BL/6N mice were purchased from Janvier (Le Genest St Isle, France) to maintain the transgenic mice in the C57BL/6N background. All mice were maintained under specific pathogen‐free conditions in the closed barrier facility of the Gene Center Munich at 23 °C, 40% humidity, and with a 12‐h light/dark cycle (lights on at 7 AM). The mice had access to water and standard rodent diet (V1534; Ssniff, Soest, Germany) *ad libitum*. Mice overexpressing LRIG1 skin specifically under the control of the keratin 5 (KRT5) promoter using the tetracycline‐controlled transcriptional activation system (TET‐Off) have been originally described previously [13]. After positive copulatory plug check, pregnant mice received 3 mg·mL^−1^ doxycycline (Dox) [Beladox 500 mg·g^−1^, bela‐pharm (Lehnecke 793‐588), Schortens, Germany] in the drinking water together with 5% sucrose (Sigma, Taufkirchen, Germany) until they gave birth to inhibit transgene expression during embryogenesis. Dissection of 8‐month‐old, female LRIG1‐TG mice (KRT5‐tTA;pTRE‐tight‐LRIG1) and controls (Co; wild‐type, KRT5‐tTA or pTRE‐tight‐LRIG1) was done at the end of the experiments; skin samples were fixed in 4% paraformaldehyde (Sigma), dehydrated, and embedded in paraffin or snap‐frozen and stored at −80 °C until use.

### 2.4 Chemical skin carcinogenesis

All mouse experiments were performed in strict compliance with the European Communities Council Directive (86/609/EEC) recommendations for the care and use of laboratory animals. The Committee on Animal Health and Care of the local governmental body of the state of Upper Bavaria (Regierung von Oberbayern), Germany, approved all mouse studies (Gz.:55.2‐1‐54‐2532‐206‐2012). Two‐stage chemical skin carcinogenesis was performed with 7‐week‐old, female LRIG1‐TG mice and controls (*n* = 14 Ctrl/11 TG) according to internationally accepted standards as described previously [30]. For tumor initiation, the carcinogen DMBA (100 µL DMBA dissolved in acetone, 400 nmol, Sigma) was applied once to the shaved back skin of LRIG1‐TG and control mice. The animals were subsequently treated twice a week with the tumor‐promoting agent TPA (50 µL TPA dissolved in ethanol, 10 nmol, Sigma) for 23 weeks. Tumor development was assessed weekly. For this purpose, the number of tumors ≥ 1 mm was counted and documented weekly, and the diameter was measured with a caliper and recorded by the same researcher every time. Papilloma burden is the calculated sum of tumor diameters per mouse.

### 2.5 Analysis of the *Hras* mutation

Genomic DNA was isolated from tail skin and tumors. Detection of the DMBA‐induced *Hras* mutation was performed as described previously [31]. Briefly, the point mutation in the *Hras* gene induces a *Xba*I restriction site in codon 61. The PCR (Qiagen, Hilden, Germany) product flanking the mutations was therefore digested with *Xba*I (Cell Signaling, Frankfurt, Germany) at 37 °C for 3 h and electrophoresed on a 3% agarose (Sigma) gel. The following primers were used: *Hras‐FW*: 5′‐AAGCCTGTTGTTTTGCAGGA‐3′; *Hras‐REV*: 5′‐GGTGGCTCACCTGTACTGATG‐3′.

### 2.6 Stimulation of A375‐LRIG1‐KO cells

For stimulation, cells were starved in the medium indicated above without FCS for 12 h and stimulation was done with 100 ng·mL^−1^ EGF (R&D Systems, Wiesbaden‐Nordenstadt, Germany) for 6 h. Three biological replicates were analyzed.

### 2.7 Western blot analysis

The Laemmli extraction buffer was used to isolate proteins from murine tissue samples. Twenty‐three weeks after tumor initiation, DMBA/TPA‐treated back skin of control and LRIG1‐TG mice without papilloma was used to analyze epidermal expression alterations due to LRIG1 excess during two‐stage chemical carcinogenesis. The protein lysis buffer [0.05 m Hepes pH 7.5, 10% glycerol, 0.15 m NaCl, 1% Triton X‐100, 0.5 m EDTA, 0.5 m EGTA, 0.01 m NaF, 0.025 m β‐glycerol phosphate, 0.01 m Na_3_Vo_4_, phosphatase inhibitor cocktail (Roche, Penzberg, Germany)] was used for protein extraction of human cell cultures. The bicinchoninic acid protein assay was used to estimate the protein concentration. 5–20 µg of total protein was separated by SDS/PAGE, transferred to PVDF membranes (Millipore, Schwalbach, Germany), and immunoblotted against antibodies as indicated. Appropriate horseradish peroxidase‐conjugated secondary antibodies were used. Immunoreactive bands were visualized by chemiluminescence with ECL kit (Millipore) and a chemiluminescence detection apparatus (ECL ChemoStar, INTAS, Göttingen, Germany). Stripping was done to analyze the phosphorylated state as well as the expression of the total protein and the reference proteins. Therefore, membranes were incubated with the stripping buffer (2% SDS, 62.5 mm Tris/HCl, pH 6.7, and 100 mm beta‐mercaptoethanol) for 40 min at 70 °C. Afterward membranes were washed, blocked, and incubated with the primary antibody. All primary and secondary antibodies and their dilutions are provided in Table S1. imagej was used for densitometrical analysis (http://rsb.info.nih.gov/ij).

### 2.8 Reverse transcriptase PCR

As previously described [32], total RNA was isolated from all investigated cell lines (HaCaT, A431, A375, SK‐MEL2, SK‐MEL28) using TRIZOL reagent (Invitrogen, Darmstadt, Germany). According to the manufacturer's instructions, 3 µg RNA was reverse‐transcribed in a final volume of 30 µL using RevertAid reverse transcriptase (Thermo Scientific, Schwerte, Germany). To show the qualitative mRNA expression of *LRIG1*, reverse transcription–PCR (RT‐PCR) was performed by using reagents from Qiagen. The final reaction volume was 20 µL, and cycle conditions were 94 °C for 5 min followed by 36 cycles of 94 °C for 1 min, 58 °C for 1 min, and 72 °C for 1 min. *GAPDH* was used as reference mRNA. The following primers were used: *hLRIG1A‐FW*: 5′‐CAAGCGGCTGATTGTTCG‐3′, *hLRIG1B‐FW*: 5′‐CAGTCGCTCACAGGACTTGG‐3′, *hLRIG1‐REV*: 5′‐CTGTGAGCGACTGATACTCC‐3′, *GAPDH‐FW*: 5′‐GTGGAAGGGCTCATGACCAC‐3′, *GAPDH‐REV*: 5′‐GCCCACAGCCTTGGCAGCA‐3′.

### 2.9 Histology, immunohistochemistry, and morphometric analysis

Skin samples were either embedded in paraffin, and 3‐µm‐thick sections were prepared using a microtome (Leica Biosystems, Wetzlar, Germany) or snap‐frozen on dry ice, embedded in Tissue‐Tek^®^ O.C.T.™ Compound (Sakura Finetek, Alphen aan den Rijn, the Netherlands), and cut into 10‐µm tissue sections using a cryostat (Microm, Walldorf, Germany). Hematoxylin and eosin (H&E) staining and immunohistochemistry were done as described previously [1]. Briefly, 10 mm sodium citrate buffer (pH 6.0) was applied for antigen retrieval (boiling, 30 min), 3% H_2_O_2_ was used to block endogenous peroxidase [room temperature (RT), 15 min], and 5% serum from the secondary antibody host in Tris‐buffered saline with 1% Tween‐20 served as blocking buffer (RT, 1 h). Bleaching was done for skin sections from the chemical carcinogenesis experiment and human melanoma samples due to the melanin accumulations. Therefore, 0.5% potassium hydroxide was added to the 3% H_2_O_2_ block and slides were incubated at 37 °C for 45 min, followed by neutralization with 1% acetic acid at RT for 20 s. Primary antibodies were incubated at 4 °C overnight; the SignalStain^®^ Boost IHC Detection Reagent (Cell Signaling, Boston, MA, USA) was used to detect primary antibodies. ImmPACT^®^ AMEC Red or 3,3′‐diaminobenzidine (Vector Laboratories) served as chromogen, and sections were counterstained with hematoxylin. All primary and secondary antibodies and their dilution are listed in Table S1. For immunofluorescence staining, the secondary antibody was conjugated to Alexa Fluor^®^ 488 and 4′,6‐diamidino‐2‐phenylindole (DAPI) was used for counterstain, blocking of the endogenous peroxidase was omitted. Morphometrical analysis was done with three different H&E stained back skin sections. Sixty pictures covering a total length of 39.2 mm of back skin epidermis were taken per animal with a 200× magnification lens using a Leica DFC425C digital camera (Leica Microsystems, Wetzlar, Germany). The LAS software version 4.4.0 (Leica Microsystems) was used to measure the area of all visible SGs and the epidermal thickness. The mean SG area was calculated and the thickness of the epidermis was determined on three constantly distributed measuring points per picture, resulting in a total of 180 measuring points per animal.

Similarly, proliferation marker protein Ki‐67 (MKI67)‐stained sections were evaluated to analyze the epidermal proliferation rate. Sixty images covering a length of 39.2 mm were used to determine the total number of epidermal nuclei and the total number of MKI67‐positive nuclei. For calculating the tumor proliferation index, the total number of nuclei and MKI67‐positive nuclei of 20 images per tumor were counted.

### 2.10 Mass spectrometry analysis

Mass spectrometry analysis was done as described previously [32]. Briefly, for mass spectrometry analysis, nonreduced protein samples of DMBA/TPA‐treated back skin of LRIG1‐TG mice and controls as well as of untreated LRIG1‐TG skin were separated by SDS/PAGE. Gels were stained with Coomassie Brilliant Blue R, and protein bands at 130 and 100 kDa were excised. To cleave disulfide bonds, gel bands were incubated in 45 mm dithioerythritol/50 mm NH_4_HCO_3_ for 30 min at 55 °C. Free sulfhydryl groups were carbamidomethylated for 2 × 15 min using iodoacetamide at a concentration of 0.1 m in 50 mm NH_4_HCO_3_ at room temperature. Prior to digestion, gel slices were minced and covered with 50 mm NH_4_HCO_3_. For digestion, 100 ng porcine trypsin (Promega, Madison, WI, USA) was added and incubated overnight. The supernatant was recovered and the peptides were further extracted from the gel with 50 mm NH_4_HCO_3_ followed by 80% acetonitrile. The pooled supernatants were dried, and the peptides were reconstituted in 0.1 formic acid. Peptides were injected on a trap column (PepMap, C18, 3 µm, 100A, 75 µm × 2 cm; Thermo Scientific, Rockford, IL, USA) and separated on a C18 column (PepMap RSLC, C18, 2 µm, 100A, 75 µm × 50 cm; Thermo Scientific) at a flow rate of 200 nL·min^−1^ using an EASY‐nLC 1000 system (Thermo Scientific). The chromatography method consisted of a gradient of 120 min from 2% to 25% B (100% acetonitrile, 0.1% formic acid) and a consecutive gradient to 50% B within 10 min. Mass spectra were acquired using a top 5 data‐dependent method on an online coupled LTQ Orbitrap XL instrument (Thermo Scientific). The acquisition method consisted of cycles of one MS (300–2000 *m/z*) followed by five data‐dependent CID MS/MS spectra at a collision energy of 35%. Spectra were searched using mascot V2.4 (Matrix Science Ltd, London, UK) and the murine subset of the UniProt database. For evaluation of the data, scaffold V 4.1 (Proteome Software, Inc, Portland, OR, USA) was used.

### 2.11 Statistical analysis

Data are presented as mean ± SEM (standard error of the mean) and compared by Student's *t*‐test (graphpad prism version 5.0 for Windows; GraphPad Software, San Diego, CA, USA). Tumor incidence was analyzed by log‐rank test, while papilloma burden and size were analyzed by 2‐way analysis of variance (ANOVA) and Student's *t*‐test. Quantitative RT‐PCR values were related to the mean value of the control group and are presented as box plots with medians. Group differences were considered to be statistically significant if *P* < 0.05.

## 3 Results

### 3.1 LRIG1 overexpression in the skin results in melanocytic tumors during chemically induced skin carcinogenesis

**Fig. 1:**
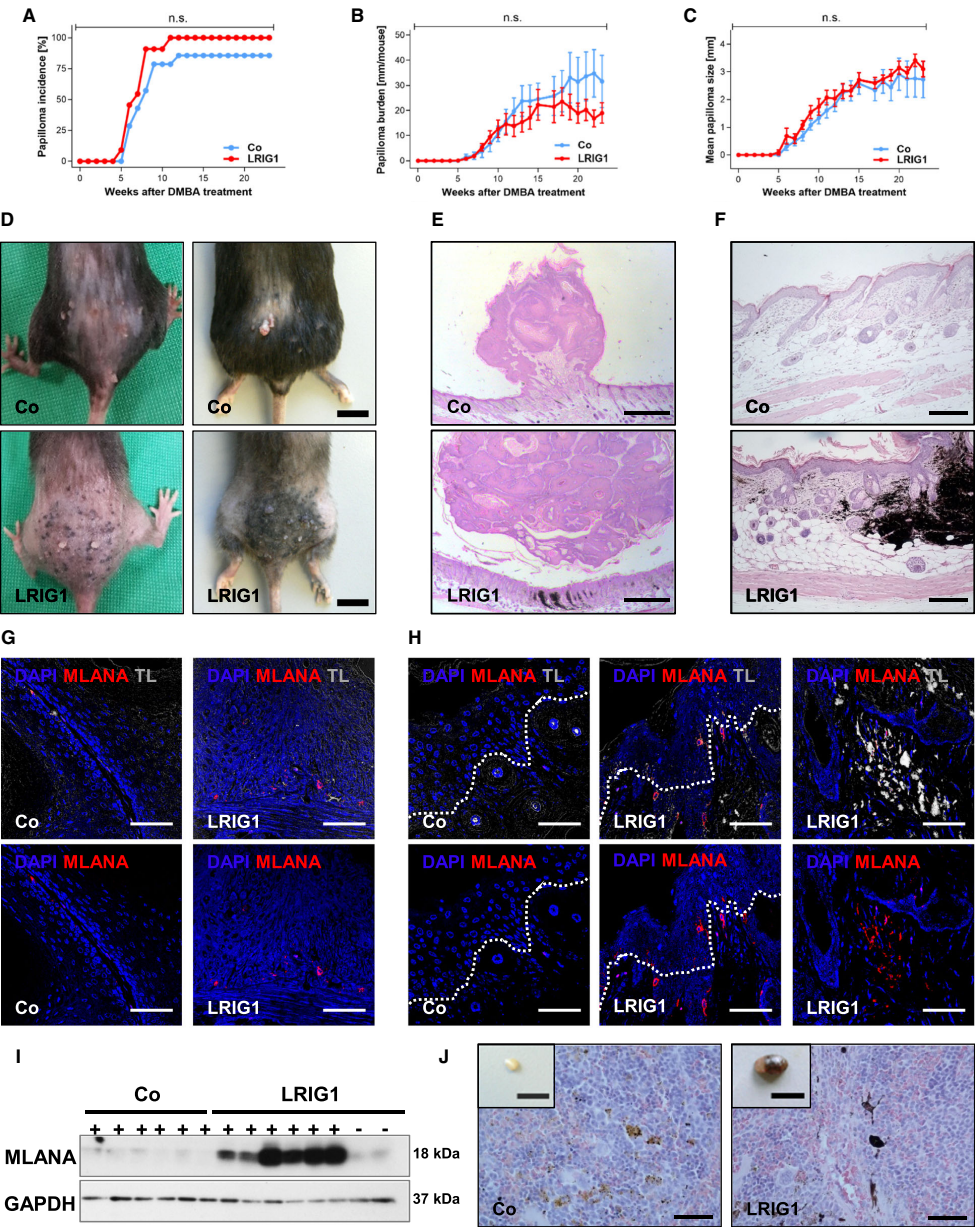
LRIG1 overexpression revealed no differences during chemical skin carcinogenesis, but causes the development of melanocytic tumors during chemical carcinogenesis. (A–C) Papilloma incidence (A), papilloma burden (B), and mean papilloma size (C) are displayed (*n* = 14 controls/11 TG). Data in (A) were analyzed by log‐rank test. Data in (B, C) were analyzed by 2‐way ANOVA and are presented as means ± SEM, and each time point was analyzed by Student's *t*‐test. (D) Representative pictures of the backs of LRIG1‐TG and control mice at 12 (left panel) and 23 (right panel) weeks after DMBA treatment. Scale bars represent 1 cm. (E) Representative H&E stainings of papillomas of LRIG1‐TG and control mice 23 weeks after DMBA treatment. Scale bars represent 1 mm. (F) H&E sections of LRIG1‐TG and control mouse back skin 23 weeks after DMBA treatment. Scale bars represent 200 µm. (G, H) Immunofluorescence labeling for MLANA (red) and DAPI (blue) on papillomas (G) and treated skin (H) of LRIG1‐TG mice and control littermates. Transmitted light is illustrated, showing melanin accumulations (white). Scale bars represent 50 µm. (I) Western blot analysis of MLANA expression in DMBA/TPA‐treated and DMBA/TPA‐untreated skin of LRIG1‐TG and control mice. GAPDH was used as reference protein. (J) Representative H&E stainings and pictures of lymph nodes of LRIG1‐TG and control mice. Scale bars represent 50 µm (H&E) and 1 cm (inlay).

We performed a two‐stage chemical skin carcinogenesis experiment to investigate if skin‐specific LRIG1 overexpression in mice affects tumor initiation or progression. Therefore, we applied a single dose of DMBA on the back skin of adult LRIG1‐TG mice and control littermates to initiate a *Hras* mutation. The inserted point mutation introduces an *Xba*I restriction site in codon 61 of the *Hras* gene and was documented in control and transgenic tumors (Fig. S1). Afterward, we promoted tumor growth by TPA treatment twice a week for 23 weeks. The first papillomas arose 5 weeks after tumor initiation in LRIG1‐TG mice and after 6 weeks in controls. The tumor incidence was comparable between TG and control animals. However, while 100% of LRIG1‐TG mice developed papillomas, only 86% of control animals were affected (Fig. 1A). In terms of papilloma burden (Fig. 1B) and papilloma size (Fig. 1C), no differences were detected between both groups. However, 12 weeks after tumor initiation, we noticed multiple black dots exclusively on the back skin of LRIG1‐TG animals, resembling melanocytic nevi (Fig. 1D). At the final stage of the experiment, all LRIG1‐TG mice (100%) developed what appeared to be heavily pigmented melanocytic tumors, covering almost the whole back (Fig. 1D). H&E staining of papillomas from LRIG1‐TG and control mice showed no morphological differences (Fig. 1E). However, histological analysis of the skin of LRIG1‐TG animals revealed dermal proliferation of heavily pigmented, spindle‐shaped melanocytes with intermixed melanophages, extending into the subcutis while control mice showed a normal skin architecture with single melanocytes at the dermal–epidermal junction (Fig. 1F). We performed an immunofluorescence labeling using melan‐A (MLANA), a melanocytic differentiation marker to identify melanocytes [33]. Our results show high expression of MLANA in papillomas (Fig. 1G) as well as in the epidermis and the dermis (Fig. 1H) of LRIG1‐TG animals and only low expression in control skin. These results were also confirmed by western blot analysis (Fig. 1I). MLANA levels in LRIG1‐TG skin were significantly increased solely during chemically induced skin carcinogenesis and not under homeostatic conditions (Fig. 1I). Moreover, the lymph nodes of LRIG1‐TG mice were enlarged and black colored compared to controls. H&E staining of lymph nodes of controls showed intermixed cells with finely dispersed melanin, whereas additionally peri‐follicular, heavily pigmented dendritic cells appeared throughout the lymph nodes of LRIG1‐TG mice (Fig. 1J). However, lymph nodes were negative for MLANA (data not shown).

### 3.2 Proliferation is significantly decreased during epidermal tumorigenesis under LRIG1 overexpression

**Fig. 2:**
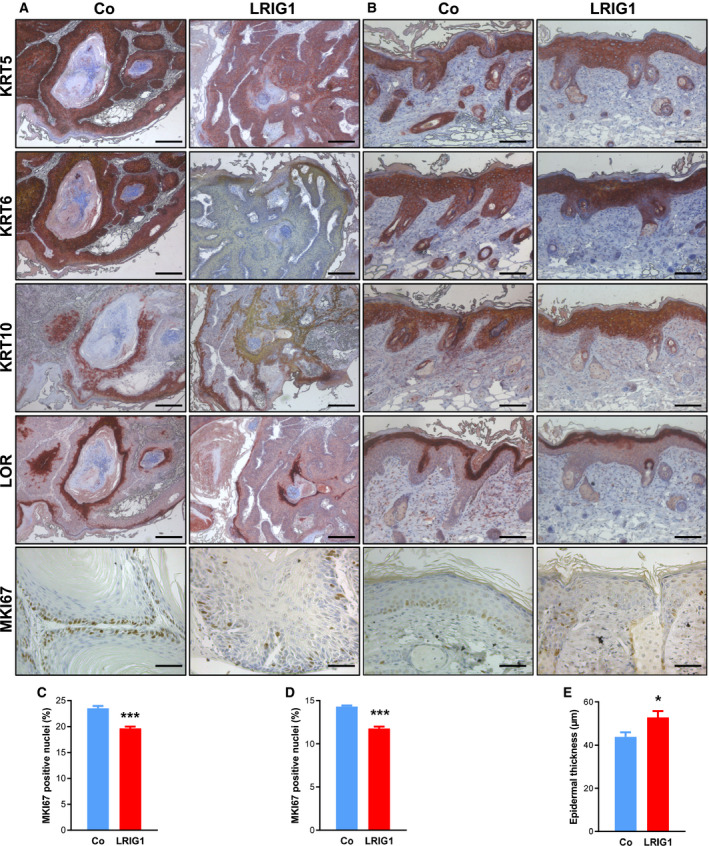
Proliferation of epidermis and papilloma is significantly decreased during epidermal tumorigenesis due to LRIG1 overexpression. (A, B) Immunostaining of paraffin‐embedded DMBA/TPA‐treated back skin of LRIG1‐TG and control animals for epidermal differentiation markers: keratin 5 (KRT5), keratin 6 (KRT6), keratin 10 (KRT10), loricrin (LOR), and the proliferation marker proliferation marker protein Ki‐67 (MKI67). Representative micrographs of a papilloma (A) and epidermis (B) of LRIG1‐TG and control mice are shown. Scale bars in (A) represent 200 µm and in (B) 100 µm. Scale bars for the MKI67 labeling represent 50 µm. (C, D) Statistical analysis of the proliferation index in papillomas (C) and the epidermis (D). (E) Morphometric analysis of epidermal thickness of LRIG1‐TG mice compared to controls. *n* = 6/group. Data are presented as means + SEM and were analyzed by Student's *t*‐test. *P < 0.05, ***P < 0.001.

To analyze the differentiation status of the tumors as well as of the IFE, we investigated the differentiation markers keratin 5, 6, 10 (KRT5, KRT6, KRT10), and loricrin (LOR) by immunohistochemistry. Labeling for KRT5, KRT10, and LOR revealed no differences between papillomas of LRIG1‐TG and control mice. However, KRT6 expression was reduced in LRIG1‐TG papillomas (Fig. 2A). No alterations in the expression pattern of the differentiation markers were found in the IFE (Fig. 2B). Additionally, we analyzed the proliferation status of papillomas as well as of the IFE by MKI67‐immunolabeling (Fig. 2A,B). Both papillomas and IFE of LRIG1‐TG mice showed a significantly decreased proliferation rate (Fig. 2C,D). The epidermal thickness of LRIG1‐TG mice was significantly increased during skin carcinogenesis (Fig. 2E).

### 3.3 LRIG1 overexpression decreased EGFR phosphorylation and increased ERBB2 activation during tumor progression

**Fig. 3:**
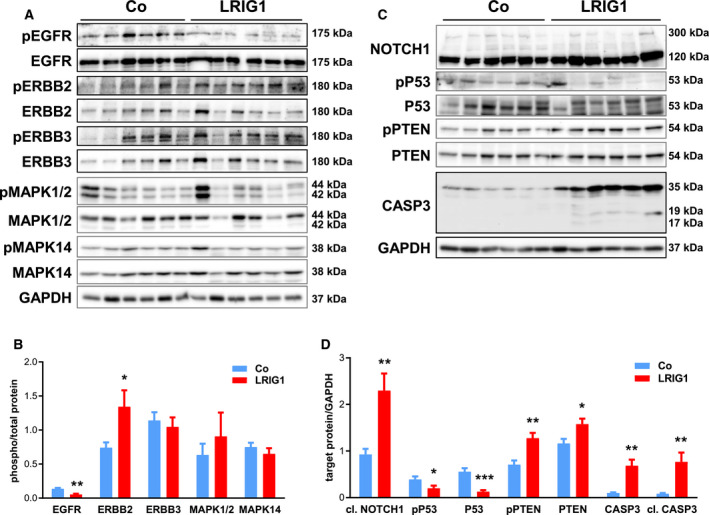
LRIG1 caused decreased EGFR activation while ERBB2 activation was increased during tumor progression. Western blots with densitometrical analyses of phosphorylated ERBB receptors and ERBB receptors, their downstream targets MAPK1/2 and MAPK14 and their phosphorylated forms (A, B), NOTCH1, CASP3, and the phosphorylated and nonphosphorylated tumor suppressors TP53 and PTEN (C, D). DMBA/TPA‐treated back skin, 23 weeks after tumor initiation, of control and LRIG1‐TG mice without papilloma were analyzed. GAPDH was used as reference protein. *n* = 6/group. Data are presented as means + SEM and were analyzed by Student's *t*‐test. *P < 0.05, **P < 0.01, ***P < 0.001.

We analyzed the influence of LRIG1 overexpression on ERBB signaling as well as on tumor suppressor proteins and apoptosis by western blot (Fig. 3). As observed for skin homeostasis [13], EGFR is significantly less phosphorylated during tumorigenesis (Fig. 3A,B). In contrast, phosphorylation of ERBB2 is significantly increased (Fig. 3A,B), as it is known from many different solid tumors [34]. Furthermore, the active, cleaved form of NOTCH1 was significantly increased in DMBA/TPA‐treated back skin under LRIG1 excess (Fig. 3C,D). Additionally, the apoptosis mediator caspase 3 (CASP3) [35] and its active form cleaved CASP3 were significantly upregulated in the skin of LRIG1‐TG mice compared to control littermates (Fig. 3C,D). Interestingly, the present results also indicated the loss of tumor suppressive function involving two independent tumor suppressors. While cellular tumor antigen p53 (TP53) and its phosphorylated form were significantly downregulated, the expression of phosphorylated phosphatidylinositol 3,4,5‐triphosphate 3‐phosphatase and dual specificity protein phosphatase PTEN (PTEN) and PTEN were upregulated in LRIG1‐TG skin during DMBA/TPA‐induced tumorigenesis (Fig. 3C,D). Thus, western blot analysis demonstrated a remarkable influence of LRIG1 excess on ERBB signaling as well as on tumorigenesis.

### 3.4 The extracellular domain of LRIG1 is extensively released during DMBA/TPA‐induced tumorigenesis

**Fig. 4:**
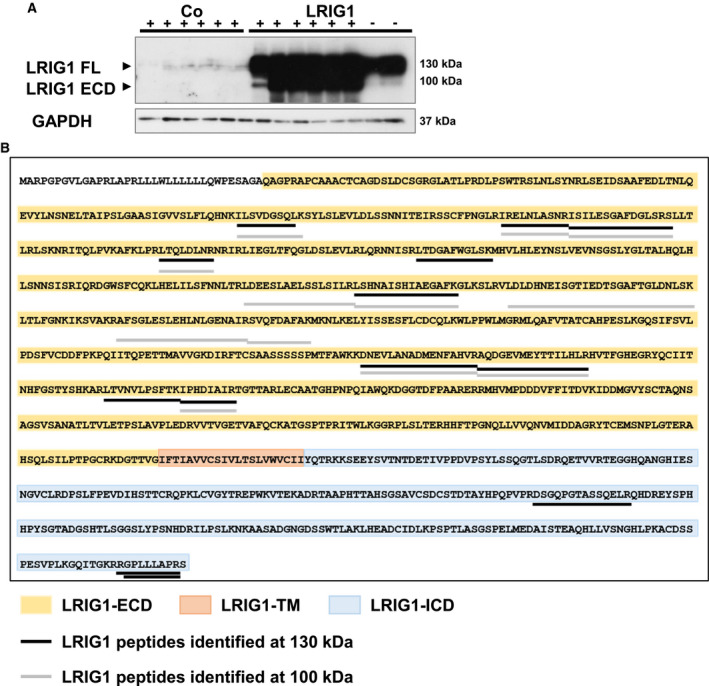
LRIG1 is shed upon DMBA/TPA treatment. (A) Western blot analysis of LRIG1 in DMBA/TPA‐treated (+) and untreated (−) skin of LRIG1‐TG and control mice. GAPDH was used as reference protein. (B) Peptides of LRIG1 protein identified by mass spectrometry. Protein samples of DMBA/TPA‐treated LRIG1‐TG back skin were separated by SDS/PAGE and bands around 130 and 100 kDa were analyzed by mass spectrometry separately. In the 100 kDa fraction (indicated in gray), only peptides of the extracellular domain of LRIG1 were identified, whereas also peptides within the intracellular domain of LRIG1 were found in the fraction around 130 kDa (indicated in black).

During the two‐stage chemical carcinogenesis, almost exclusively LRIG1‐TG mice developed melanocytic tumors starting 12 weeks after tumor initiation. However, the used KRT5 promoter drives LRIG1 expression in keratinocytes of the basal layer of the IFE and HFs, but not in melanocytes. As melanocytes are restricted to the basal epidermis and HFs, we assumed a paracrine mechanism for LRIG1 function. The western blot in Fig. 4A shows that LRIG1 was shed during DMBA/TPA‐induced carcinogenesis. We detected the full‐length protein of LRIG1 (LRIG1‐FL) at 130 kDa and the extracellular domain of LRIG1 (LRIG1‐ECD) at 100 kDa (Fig. 4A). Remarkably, LRIG1 was cleaved from the keratinocyte surface only upon DMBA/TPA treatment. Without treatment, LRIG1‐TG mice showed only minor cleavage (Fig. 4A). To confirm the presence of the different protein domains of LRIG1, we performed mass spectrometry analysis of the excised protein bands. In the 130 kDa fractions of DMBA/TPA‐treated and DMBA/TPA‐untreated TG samples, we detected peptides specific for the ECD and the intracellular domain of LRIG1, whereas no LRIG1 peptides were found in the treated control sample. In the 100 kDa fractions, we detected peptides exclusively from the ECD of LRIG1 in TG samples. Figure 4B shows all identified LRIG1 peptides in the 130 kDa fraction (black) and the 100 kDa fraction (gray) in DMBA/TPA‐treated LRIG1‐TG back skin. Figure S2 gives an overview of all identified LRIG1 peptides in the different fractions and Table S2 provides the detailed mass spectrometry data. Altogether, these observations indicate that LRIG1 is extensively shed during tumorigenesis allowing soluble ECD of LRIG1 to interact with surrounding melanocytes.

### 3.5 LRIG1 is expressed in human skin cancer

**Fig. 5:**
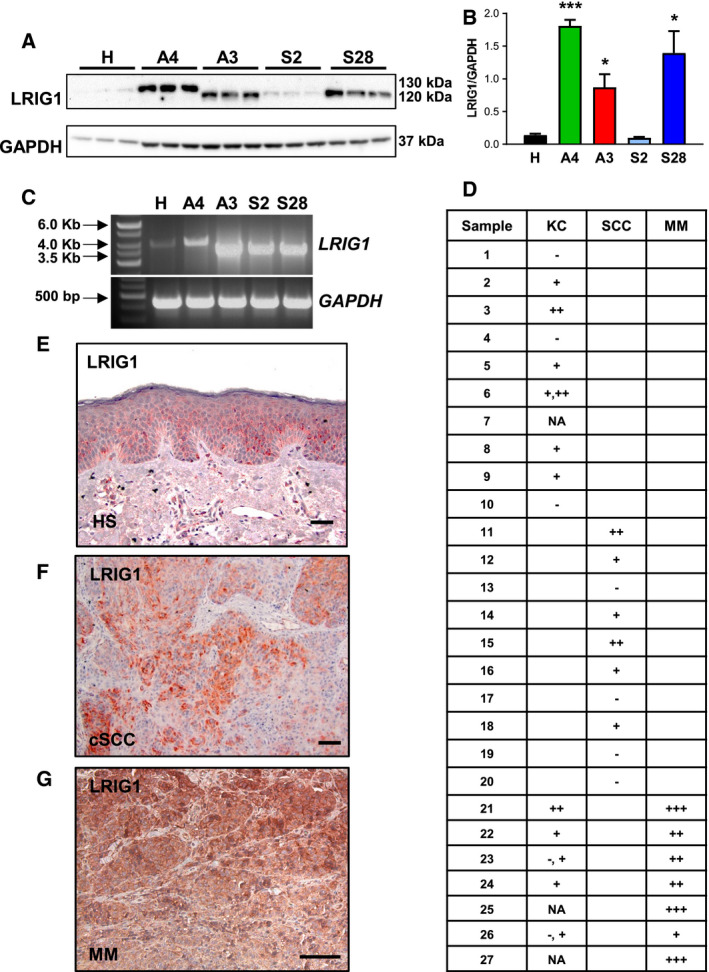
LRIG1 is expressed in human skin, cutaneous squamous cell carcinoma, and melanoma. (A, B) Western blot (A) and densitometrical (B) analysis of LRIG1 expression in HaCaT, A431, A375, SK‐MEL2, and SK‐MEL28 cell lines. LRIG1‐A/B indicates LRIG1 isoform A/B. GAPDH was used as reference protein. *n* = 3/group. Data are presented as means + SEM and were analyzed by Student's *t*‐test. *P < 0.05, ***P < 0.001. (C) RT‐PCR showing *LRIG1* isoform B in HaCaT and A431 cells and isoform A in A375, SK‐MEL2, and SK‐MEL28 cell lines. *GAPDH* was used as reference gene. (E–G) Immunohistochemical labeling on paraffin‐embedded human cancer tissue sections of healthy human skin (E), cSCC (F), and melanoma (G) patients for LRIG1. HS, healthy skin; MM, malignant melanoma; cSCC, cutaneous squamous cell carcinoma. Scale bars represent 100 µm. (D) Evaluation of LRIG1 immunoreactivity intensity in samples of 10 healthy individuals, 10 cSCC patients, and seven malignant melanoma patients. KC, keratinocytes; −, negative for LRIG1; +, weak immunoreactivity; ++, middle immunoreactivity, +++, strong immunoreactivity; N.A., not applicable.

LRIG1 influences mouse skin carcinogenesis and plays a role in the development of human cSCC and melanoma, where LRIG1 expression appears to be a prognostic factor for a good prognosis [23, 24, 36]. We detected LRIG1 expression by western blot analysis in different human cell lines of epidermal origin, including HaCaT keratinocytes, cSCC cells, (A431), and various melanoma cell lines (A375, SK‐MEL2, SK‐MEL28) (Fig. 5A). LRIG1 expression was significantly increased in three of four human skin cancer cell lines compared to HaCaT keratinocytes: A431, A375, and SK‐MEL28 (Fig. 5A,B). Interestingly, we identified two different LRIG1 isoforms: HaCaT keratinocytes and A431 cSCC cells expressed isoform A of LRIG1, whereas all melanoma cell lines expressed the smaller isoform B (Fig. 5A). The presence of the two different isoforms of *LRIG1* was confirmed by RT‐PCR (Fig. 5C) and sequencing technology (Figs S3 and S4). Isoform B has an additional exon 10 with 24 amino acids (aa), and 47 aa of exon 15 are missing (Fig. S5). Both exons code leucine‐rich repeats in the extracellular domain of LRIG1 and slightly change the aa sequence in these areas. Additionally, we analyzed LRIG1 expression in human tissue samples of healthy individuals and patients with cSCC or malignant melanoma by immunohistochemistry (Fig. 5D–G). The relative staining intensity was graded by comparison it with that of healthy epidermis, which was used as positive control. In the majority of normal human skin samples, LRIG1 was mainly expressed in the lower spinous layers of the IFE and the infundibulum of HFs (data not shown). The staining was mainly cytoplasmic, but some nuclei in the spinous layer were also positive (Fig. 5E). Immunohistochemical analyses revealed weak‐to‐medium expression of LRIG1 in differentiated areas in 6 out of 10 cSCCs, while 4 turned out to be entirely negative. Only in two cases (sample 1 and sample 5), we could see a direct increase in the intensity of LRIG1 immunoreactivity between cSCC patient samples and healthy skin (Fig. 5D; Fig. S8a). Six melanoma samples displayed a high LRIG1 protein expression and only one sample showed a medium LRIG1 protein expression. Four melanoma samples revealed an increased immunoreactivity compared with healthy skin (Fig. 5D; Fig. S8b).

### 3.6 Loss of LRIG1 causes ERBB receptor downregulation upon EGF stimulation

**Fig. 6:**
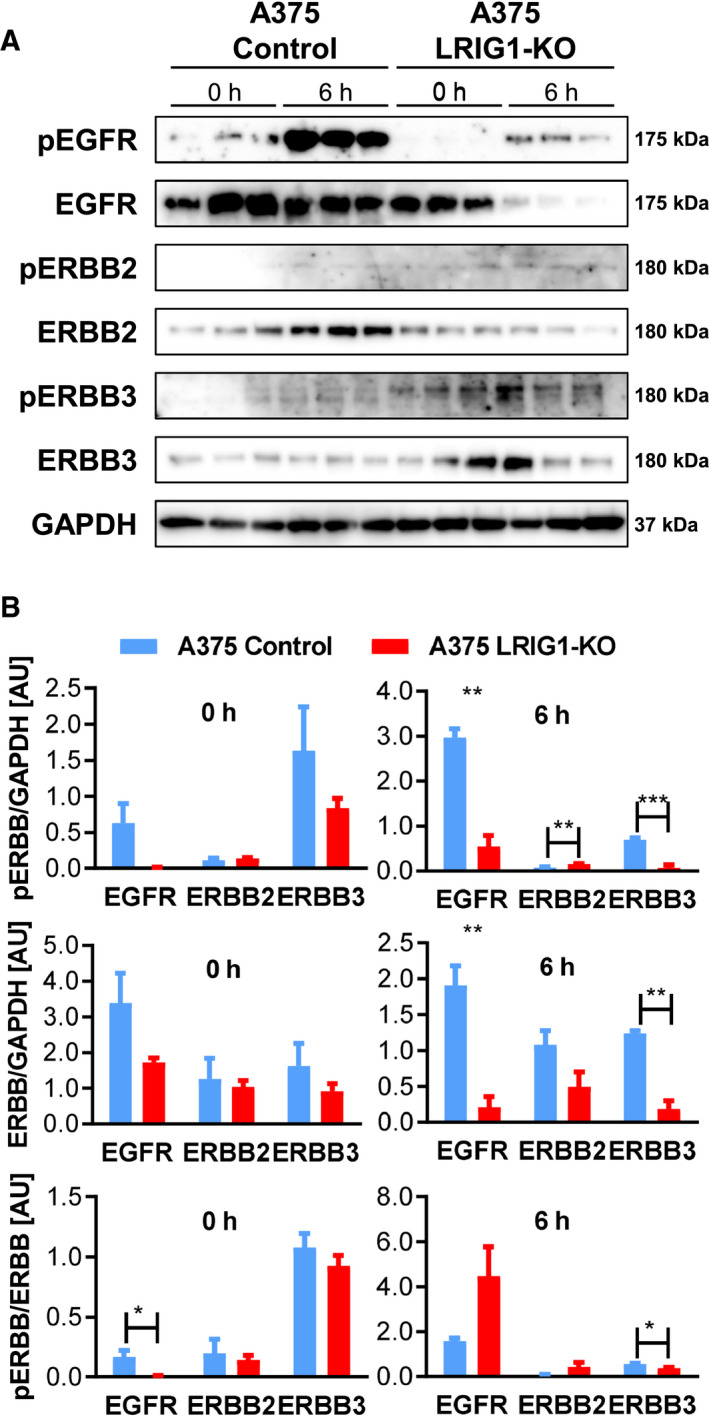
Loss of LRIG1 causes EGFR and ERBB3 downregulation upon EGF stimulation in A375 melanoma cells. Western blot (A) and densitometrical (B) analysis of phosphorylated ERBB receptors and ERBB receptors in A375 control and A375‐ LRIG1‐KO cells unstimulated and 6 h after EGF stimulation. GAPDH was used as reference protein. *n* = 3/group. Data are presented as means + SEM and were analyzed by Student's *t*‐test. *P < 0.05, **P < 0.01, ***P < 0.001.

For further studies, we focused on the human melanoma cell line A375. As A375 melanoma cells revealed high intrinsic LRIG1 expression, we deleted LRIG1 via CRISPR/Cas9‐mediated gene editing to investigate its function in A375 cells and its influences on the ERBB receptor network. The strategy applied and the identified clones are shown in Fig. S6. Compared to controls, we did not see any differences with respect to ERBB receptor expression in A375‐LRIG1‐KO cells except a significant downregulation of EGFR (Fig. 6A,B). Since LRIG1 is known to be a negative regulator of ERBBs, we stimulated cells with EGF and analyzed changes in the expression and phosphorylation of ERBB receptors in the absence of LRIG1. In A375 control cells, LRIG1 expression was constantly high and EGFR phosphorylation was lost 9 h after EGF stimulation, and in HaCaT keratinocytes, we observed LRIG1 upregulation upon EGF stimulation after 6 h (Fig. S7). Therefore, we stimulated A375 control and LRIG1‐KO cells for 6 h and investigated the influence of the loss of LRIG1 on ERBB receptor expression and activation. The deletion of LRIG1 in A375 cells caused a significant decrease in ERBB3 activation 6 h after EGF stimulation (Fig. 6A,B). The phosphorylation of EGFR and ERBB3 was significantly decreased, whereas ERBB2 phosphorylation was increased in A375‐LRIG1‐KO cells compared to A375 controls (Fig. 6A,B). Additionally, ERBB receptor expression was decreased after 6 h EGF stimulation, but only the decrease in EGFR and ERBB3 expression was significant (Fig. 6A,B). Thus, the deletion of LRIG1 showed intriguing effects on ERBB receptors in A375 melanoma cells, considerably decreasing ERBB receptor expression.

## 4 Discussion

Receptor tyrosine kinases play an important role in skin cancer development and progression and therefore could be promising targets for therapy. Unfortunately, RTK inhibitors often show severe side effects; the identification of new and more specific targets might thus contribute to improved treatment strategies [8]. In this regard, we focused on LRIG proteins (LRIG1‐3), which are regulators of RTKs such as the ERBB receptor family [37]. LRIG proteins attracted attention due to their implication as prognostic factors in various tumor types [20]. While LRIG1 and LRIG3 are thought to be tumor suppressors, LRIG2 expression is mostly related to poor prognosis [38]. Previously, we have shown that LRIG2 acts as an oncogene in skin cancer affecting ERBB signaling [32]. In this study, we used a LRIG1‐TG gain‐of‐function mouse model to investigate the influence of LRIG1 overexpression on skin tumorigenesis. Compared to control mice, LRIG1‐TG mice showed no alteration during chemically induced skin carcinogenesis, but displayed a decreased proliferation rate of papillomas. cSCC is usually characterized by high KRT6 expression levels [39], and KRT6 was detected in the IFE of DMBA/TPA‐treated skin in both groups but was decreased in papillomas of LRIG1‐TG mice. While differentiation of the IFE of LRIG1‐TG mice was not affected, epidermal thickness was increased and the proliferation was significantly decreased. Under homeostatic conditions, LRIG1‐TG mice show also thickened epidermis but increased proliferation rate [13]. Consequently, the DMBA/TPA treatment may cause a less pronounced increase in epidermal thickness, which is in line with the decreased proliferation rate. Notably, LRIG1‐TG mice developed melanocytic tumors, which were visible 12 weeks after tumor initiation. Additionally, the melanocyte differentiation marker, MLANA, was highly upregulated in DMBA/TPA‐treated back skin of LRIG1‐TG mice compared to untreated LRIG1‐TG and treated control animals. However, using the Tet‐Off system with the KRT5 promoter, LRIG1 excess only appears in keratinocytes of the epidermal basal layer. Yi *et al*. [40] showed that LRIG1 can be shed from the cell surface and also acts in a non‐cell‐autonomous mechanism. Therefore, we assessed a possible paracrine effect of LRIG1 on melanocytes by studying the presence of the LRIG1‐ECD by western blot and mass spectrometry. While the DMBA/TPA‐treated back skin of LRIG1‐TG animals showed expression of LRIG1‐FL and LRIG1‐ECD, untreated LRIG1‐TG mice revealed only a weak cleavage of LRIG1. LRIG1 expression in DMBA/TPA‐treated control skin was too low to be detected. It is known that TPA induces ECD shedding of diverse cell surface proteins [41] and we cannot exclude that the cleavage of the LRIG1‐ECD may occur due to the TPA treatment. However, the ECD of LRIG1 may influence neighboring melanocytes, inducing the production of melanin and the development of melanocytic nevi. A high number of nevi is a known risk factor for the development of malignant melanomas which may reflect a potential oncogenic function of LRIG1 in the skin [42]. In contrast, it was reported that an overexpression of LRIG1 decreased hypoxia‐induced invasion, migration, and vasculogenic mimicry in human A2058 melanoma cells, thus acting as a tumor suppressor [36]. We also analyzed the influence of LRIG1 excess on EGFR/ERBB receptors during epidermal tumorigenesis. While EGFR activity was still decreased after the two‐stage chemical carcinogenesis in LRIG1‐TG mice compared to controls, the activation of ERBB2 was significantly increased. ERBB2 is often upregulated in various cancers [34] and in the skin ERBB2 is essential for tumor progression [2]; its excess induces epidermal tumor initiation and spontaneous formation of papillomas [43]. Moreover, the ERBB2/ERBB3 complex seems to be a promising target for combination tumor therapy in cutaneous melanoma [44], and activated NOTCH1 and ERBB2/ERBB3 signaling are involved in melanoma pathogenesis [45], supporting our data. The downregulation of the tumor suppressor TP53 and the increase of inactive PTEN, during carcinogenesis also point to an oncogenic function of epidermal LRIG1 excess.

To evaluate whether these unexpected findings are of human relevance, we analyzed different human skin cell lines and tissue samples of healthy individuals and patients with cSCC or melanoma. Immunohistochemical labeling showed that LRIG1 is expressed in malignant melanoma as well as in cSCC. As LRIG1 is a marker for epidermal progenitor cells, its expression was very low and barely detectable in the HaCaT cell line but was significantly upregulated in three of the four skin cancer cell lines. The melanoma cell lines A375, SK‐MEL2, and SK‐MEL28 expressed LRIG1 isoform A, whereas HaCaT keratinocytes and A431 cells expressed isoform B. The expression of different isoforms of LRIG1 in keratinocytes and melanocytes may refer to context‐specific functions, which might explain an oncogenic impact on tumorigenesis as well as tumor suppressor activity in other tissues [21, 22, 23, 24, 46]. The loss of LRIG1 in A375 melanoma cells resulted in decreased expression of ERBB receptors upon EGF stimulation and decreased activation of ERBB3. ERBB3 plays an important role during melanoma development and contributes to poor survival of melanoma patients with metastases [47]. Consequently, our findings indicate a tumorigenic function of LRIG1 also in humans, as its deletion led to ERBB3 downregulation upon EGF stimulation in A375 cells.

## 5 Conclusion

In summary, LRIG1 overexpression caused no alterations during chemically induced skin carcinogenesis, but promoted the formation of multiple melanocytic tumors. Additionally, the deletion of LRIG1 in human melanoma cells led to the downregulation of the ERBB receptor network and uncovered LRIG1 as a potential oncogene in melanoma. Thus, the present study revealed a new tumorigenic function of LRIG1 during mouse epidermal carcinogenesis as well as potentially in human keratinocytes and more particularly in melanoma cells.

## Conflict of interest

The authors declare no conflict of interest.

## Author contributions

MD and CH contributed to conception and design; MD and CH contributed to development of methodology; MD, MG, and CP acquired the data; MD, CH, HK, TF, and CP made analysis and interpretation of data; MD, CH, and MRS involved in writing, review, and/or revision of the manuscript; MD supervised the study; and MD, CH, MRS, MG, HK, TF, and CP made discussion of the experiments at planning stage and discussions of the results.

### Peer Review

The peer review history for this article is available at https://publons.com/publon/10.1002/1878‐0261.12945.

## Supporting information


**Fig. S1.** Analysis of DMBA‐induced *Hras* mutation.

**Fig. S2.** Mass spectrometry data of LRIG1‐TG back skin.

**Fig. S3.** cDNA sequence of human *LRIG1* isoform A.

**Fig. S4.** cDNA sequence of human *LRIG1* isoform B.

**Fig. S5.** Amino acid sequence comparison of human LRIG1 isoforms.

**Fig. S6.** Generation of A375 LRIG1 knockout cell lines.

**Fig. S7.** Western blots of HaCaT and A375 cell lines.

**Fig. S8.** Immunohistochemical labeling of LRIG1 in human cSCC and melanoma.

**Table S1.** Antibodies employed for Western blots analysis, immunoprecipitation, immunohistochemistry, and immunofluorescence.

**Table S2.** Total spectrum counts of LRIG1 peptides.

## Data Availability

All data generated or analyzed during this study are included in this published article and its Supporting information files.
