# Supplementary material for: The transmembrane protein LRIG1 triggers melanocytic tumor development following chemically induced skin carcinogenesis
Source: Mol Oncol. 2021 Mar 31;15(8):2140–55. doi: 10.1002/1878-0261.12945 (PMC8495683; doi:10.1002/1878-0261.12945)
Supplement: Supplementary file 1 — Fig. S1. Analysis of DMBA‐induced Hras mutation. [file MOL2-15-2140-s005.pdf]

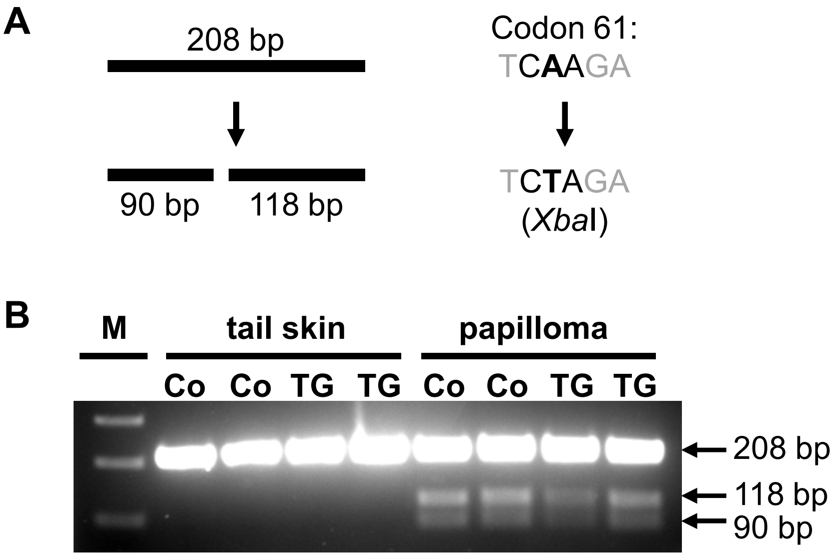

Suplementary Figure S1. (A) Scheme to detect the DMBA-induced *Hras* mutation in codon 61 (C**A**A). A 208-bp fragment flanking the *Hras* codon 61 was amplified by PCR and subjected to an *Xba*I digest to detect the A¡T transversion (C**T**A). On position 61 amino acid glutamine will be replaced by leucine. (B) The *Hras* mutation is present in back skin papilloma, tail skin was used as negative control. Electrophoresis of digestion a 3% agarose gel. M: DNA marker; Co: control; TG: LRIG1-TG; bp: base pairs.

Hoesl et al.
Fig. S1
