# Supplementary material for: The transmembrane protein LRIG1 triggers melanocytic tumor development following chemically induced skin carcinogenesis
Source: Mol Oncol. 2021 Mar 31;15(8):2140–55. doi: 10.1002/1878-0261.12945 (PMC8495683; doi:10.1002/1878-0261.12945)
Supplement: Supplementary file 2 — Fig. S2. Mass spectrometry data of LRIG1‐TG back skin. [file MOL2-15-2140-s008.pdf]

A

P70193 (100%), 119,159.1 Da
Leucine-rich repeats and immunoglobulin-like domains protein 1 OS=Mus musculus OX=10090 GN=Lrig1 PE=1 SV=2
13 exclusive unique peptides, 13 exclusive unique spectra, 13 total spectra, 143/1091 amino acids (13% coverage)

MARPGPGVLG APRLAPRLLL WLLLLLLQWP ESAGAQAGPR APCAAACTCA GDSLDCSGRG LATLPRDLPS WTRSLNLSYN
RLSEIDSAAF EDLTNLQEVY LNSNELTAIP SLGAASIGVV SLFLQHNK**IL** **SVDGSQLK**SY LSLEVLDLSS NNITEIRSSC
FPNGLR**IREL** **NLASNRISIL** **ESGAFDGLSR** SLLTLRLSKN RITQLPVKAF KLPR**LTQLDL** **NR**NRIRLIEG LTFQGLDSLE
VLRLQRNNIS R**LTDGAFWGL** **SK**MHVLHLEY NSLVEVNSGS LYGLTALHQL HLSNNSISRI QRDGWSFCQK LHELILSFNN
LTRLDEESLA ELSSLSILR**L** **SHNAISHIAE** **GAFK**GLKSLR VLDLDHNEIS GTIEDTSGAF TGLDNLSKLT LFGNKIKSVA
KRAFSGLESL EHLNLGENAI RSVQFDAFAK MKNLKELYIS SESFLCDCQL KWLPPWLMGR MLQAFVTATC AHPESLKGQS
IFSVLPDSFV CDDFPKPQII TQPETTMAVV GKDIRFTCSA ASSSSSPMTF AWKK**DNEVLA** **NADMENFAHV** **RAQDGEVMEY**
TTILHLRHVT FGHEGRYQCI ITNHFGSTYS HKAR**LTVNVL** **PSFTKIPHDI** **AIR**TGTTARL ECAATGHPNP QIAWQKDGGT
DFPAARERRM HVMPDDDVFF ITDVKIDDMG VYSCTAQNSA GSVSANATLT VLETPSLAVP LEDRVVTVGE TVAFQCKATG
SPTPRITWLK GGRPLSLTER HHFTPGNQLL VVQNVMIDDA GRYTCEMSNP LGTERAHSQL SILPTPGCRK DGTTVGIFTI
AVVCSIVLTS LVWVCIIYQT RKKSEEYSVT NTDETIVPPD VPSYLSSQGT LSDRQETVVR TEGGHQANGH IESNGVCLRD
PSLFPEVDIH STTCRQPKLC VGYTREPWKV TEKADRTAAP HTTAHSGSAV CSDCSTDTAY HPQPVPR**DSG** **QPGTASSQEL**
RQHDREYSPH HPYSGTADGS HTLSGGSLYP SNHDRILPSL KNKAASADGN GDSSWTLAKL HEADCIDLKP SPTLASGSPE
LMEDAISTEA QHLLVSNGHL PKACDSSPES VPLKGQITGK R**RGPLLLAPR** S

B

P70193 (100%), 119,159.1 Da
Leucine-rich repeats and immunoglobulin-like domains protein 1 OS=Mus musculus OX=10090 GN=Lrig1 PE=1 SV=2
12 exclusive unique peptides, 12 exclusive unique spectra, 12 total spectra, 170/1091 amino acids (16% coverage)

MARPGPGVLG APRLAPRLLL WLLLLLLQWP ESAGAQAGPR APCAAACTCA GDSLDCSGRG LATLPRDLPS WTRSLNLSYN
RLSEIDSAAF EDLTNLQEVY LNSNELTAIP SLGAASIGVV SLFLQHNK**IL** **SVDGSQLK**SY LSLEVLDLSS NNITEIRSSC
FPNGLR**IREL** **NLASNRISIL** **ESGAFDGLSR** SLLTLRLSKN RITQLPVKAF KLPR**LTQLDL** **NR**NRIRLIEG LTFQGLDSLE
VLRLQRNNIS RLTDGAFWGL SKMHVLHLEY NSLVEVNSGS LYGLTALHQL HLSNNSISRI QRDGWSFCQK LHELILSFNN
LTR**LDEESLA** **ELSSLSILRL** **SHNAISHIAE** **GAFK**GLKSLR **VLDLDHNEIS** **GTIEDTSGAF** **TGLDNLSK**LT LFGNKIKSVA
KR**AFSGLESL** **EHLNLGENAI** **RSVQFDAFAK** MKNLKELYIS SESFLCDCQL KWLPPWLMGR MLQAFVTATC AHPESLKGQS
IFSVLPDSFV CDDFPKPQII TQPETTMAVV GKDIRFTCSA ASSSSSPMTF AWKK**DNEVLA** **NADMENFAHV** **RAQDGEVMEY**
TTILHLRHVT FGHEGRYQCI ITNHFGSTYS HKARLTVNVL PSFTK**IPHDI** **AIR**TGTTARL ECAATGHPNP QIAWQKDGGT
DFPAARERRM HVMPDDDVFF ITDVKIDDMG VYSCTAQNSA GSVSANATLT VLETPSLAVP LEDRVVTVGE TVAFQCKATG
SPTPRITWLK GGRPLSLTER HHFTPGNQLL VVQNVMIDDA GRYTCEMSNP LGTERAHSQL SILPTPGCRK DGTTVGIFTI
AVVCSIVLTS LVWVCIIYQT RKKSEEYSVT NTDETIVPPD VPSYLSSQGT LSDRQETVVR TEGGHQANGH IESNGVCLRD
PSLFPEVDIH STTCRQPKLC VGYTREPWKV TEKADRTAAP HTTAHSGSAV CSDCSTDTAY HPQPVPRDSG QPGTASSQEL
RQHDREYSPH HPYSGTADGS HTLSGGSLYP SNHDRILPSL KNKAASADGN GDSSWTLAKL HEADCIDLKP SPTLASGSPE
LMEDAISTEA QHLLVSNGHL PKACDSSPES VPLKGQITGK RRGPLLLAPR S

C

P70193 (100%), 119,159.1 Da
Leucine-rich repeats and immunoglobulin-like domains protein 1 OS=Mus musculus OX=10090 GN=Lrig1 PE=1 SV=2
5 exclusive unique peptides, 5 exclusive unique spectra, 5 total spectra, 69/1091 amino acids (6% coverage)

MARPGPGVLG APRLAPRLLL WLLLLLLQWP ESAGAQAGPR APCAAACTCA GDSLDCSGRG LATLPRDLPS WTRSLNLSYN
RLSEIDSAAF EDLTNLQEVY LNSNELTAIP SLGAASIGVV SLFLQHNKIL SVDGSQLKSY LSLEVLDLSS NNITEIRSSC
FPNGLRIREL NLASNR**ISIL** **ESGAFDGLSR** SLLTLRLSKN RITQLPVKAF KLPR**LTQLDL** **NR**NRIRLIEG LTFQGLDSLE
VLRLQRNNIS R**LTDGAFWGL** **SK**MHVLHLEY NSLVEVNSGS LYGLTALHQL HLSNNSISRI QRDGWSFCQK LHELILSFNN
LTRLDEESLA ELSSLSILRL SHNAISHIAE GAFKGLKSLR **VLDLDHNEIS** **GTIEDTSGAF** **TGLDNLSK**LT LFGNKIKSVA
KRAFSGLESL EHLNLGENAI RSVQFDAFAK MKNLKELYIS SESFLCDCQL KWLPPWLMGR MLQAFVTATC AHPESLKGQS
IFSVLPDSFV CDDFPKPQII TQPETTMAVV GKDIRFTCSA ASSSSSPMTF AWKKDNEVLA NADMENFAHV RAQDGEVMEY
TTILHLRHVT FGHEGRYQCI ITNHFGSTYS HKARLTVNVL PSFTKIPHDI AIRTGTTARL ECAATGHPNP QIAWQKDGGT
DFPAARERRM HVMPDDDVFF ITDVKIDDMG VYSCTAQNSA GSVSANATLT VLETPSLAVP LEDRVVTVGE TVAFQCKATG
SPTPRITWLK GGRPLSLTER HHFTPGNQLL VVQNVMIDDA GRYTCEMSNP LGTERAHSQL SILPTPGCRK DGTTVGIFTI
AVVCSIVLTS LVWVCIIYQT RKKSEEYSVT NTDETIVPPD VPSYLSSQGT LSDRQETVVR TEGGHQANGH IESNGVCLRD
PSLFPEVDIH STTCRQPKLC VGYTREPWKV TEKADRTAAP HTTAHSGSAV CSDCSTDTAY HPQPVPRDSG QPGTASSQEL
RQHDREYSPH HPYSGTADGS HTLSGGSLYP SNHDRILPSL KNKAASADGN GDSSWTLAKL HEADCIDLKP SPTLASGSPE
LMEDAISTEA QHLLVSNGHL PKACDSSPES VPLKGQITGK RR**GPLLLAPR** S

D

P70193 (99%), 119,159.1 Da
Leucine-rich repeats and immunoglobulin-like domains protein 1 OS=Mus musculus OX=10090 GN=Lrig1 PE=1 SV=2
1 exclusive unique peptides, 1 exclusive unique spectra, 1 total spectra, 8/1091 amino acids (1% coverage)

MARPGPGVLG APRLAPRLLL WLLLLLLQWP ESAGAQAGPR APCAAACTCA GDSLDCSGRG LATLPRDLPS WTRSLNLSYN
RLSEIDSAAF EDLTNLQEVY LNSNELTAIP SLGAASIGVV SLFLQHNKIL SVDGSQLKSY LSLEVLDLSS NNITEIRSSC
FPNGLRIREL NLASNRISIL ESGAFDGLSR SLLTLRLSKN RITQLPVKAF KLPR**LTQLDL** **NR**NRIRLIEG LTFQGLDSLE
VLRLQRNNIS RLTDGAFWGL SKMHVLHLEY NSLVEVNSGS LYGLTALHQL HLSNNSISRI QRDGWSFCQK LHELILSFNN
LTRLDEESLA ELSSLSILRL SHNAISHIAE GAFKGLKSLR VLDLDHNEIS GTIEDTSGAF TGLDNLSKLT LFGNKIKSVA
KRAFSGLESL EHLNLGENAI RSVQFDAFAK MKNLKELYIS SESFLCDCQL KWLPPWLMGR MLQAFVTATC AHPESLKGQS
IFSVLPDSFV CDDFPKPQII TQPETTMAVV GKDIRFTCSA ASSSSSPMTF AWKKDNEVLA NADMENFAHV RAQDGEVMEY
TTILHLRHVT FGHEGRYQCI ITNHFGSTYS HKARLTVNVL PSFTKIPHDI AIRTGTTARL ECAATGHPNP QIAWQKDGGT
DFPAARERRM HVMPDDDVFF ITDVKIDDMG VYSCTAQNSA GSVSANATLT VLETPSLAVP LEDRVVTVGE TVAFQCKATG
SPTPRITWLK GGRPLSLTER HHFTPGNQLL VVQNVMIDDA GRYTCEMSNP LGTERAHSQL SILPTPGCRK DGTTVGIFTI
AVVCSIVLTS LVWVCIIYQT RKKSEEYSVT NTDETIVPPD VPSYLSSQGT LSDRQETVVR TEGGHQANGH IESNGVCLRD
PSLFPEVDIH STTCRQPKLC VGYTREPWKV TEKADRTAAP HTTAHSGSAV CSDCSTDTAY HPQPVPRDSG QPGTASSQEL
RQHDREYSPH HPYSGTADGS HTLSGGSLYP SNHDRILPSL KNKAASADGN GDSSWTLAKL HEADCIDLKP SPTLASGSPE
LMEDAISTEA QHLLVSNGHL PKACDSSPES VPLKGQITGK RRGPLLLAPR S

Supplementary Figure S2: LRIG1 peptides identified by mass spectrometry in DMBA/TPA-treated back skin of LRIG1-TG skin at 130 kDa (A) and 100 kDa (B) and of untreated LRIG1-TG skin at 130 kDa (C) and 100 kDa (D).

Hoesl et al.
Fig. S2
