# Supplementary material for: The transmembrane protein LRIG1 triggers melanocytic tumor development following chemically induced skin carcinogenesis
Source: Mol Oncol. 2021 Mar 31;15(8):2140–55. doi: 10.1002/1878-0261.12945 (PMC8495683; doi:10.1002/1878-0261.12945)
Supplement: Supplementary file 3 — Fig. S3. cDNA sequence of human LRIG1 isoform A. [file MOL2-15-2140-s001.pdf]

CAAGCGGCTGATTGTTCGGCTGCGAGGTCGGCGGGACTCTTGCTGCGCGGAGGAGCCGTGCGGAACCCGAGGCTCGCGCGCGCGC
GCGCTCTCTGGCCGCGGCGTGGGGACAGCGAGGCGCACTGGGGCCTCCCAGCGCGGGGCCGGCCGCGCCGTCCAGCCCGAGGTCT
ACGGCTTTGCGCTCCGAGCCCAGAGGAAGATGCCTGCCGGCACCGAGCTCGGGCTGCGGGGCTGAACGCCTGTCTTCCAGGCGCA
GCGGCAGCACTGCCCTCGGCCGGTGTCGGTAGCGGCACTCGGCGTGCCCCGGGCGGACGAAGAGCGCAGGCTGGGTACACCTTGC
CCGAATCGGCGGAGTTCGCAGCTAGCGCGGGCGGGCCGGCCGGCCCGGATGGGCGCGGGGTTTGCGGCCCCCGCCGGGTGCTCCG
GAGCGGCCCGGGCACCGGGGGCACGCTGAGTGCCGGAGCCGCGGCCGCAGAGAGAACTTGGGGCGGGGGCCATGCCCCGGTGCGG
AGTCTAGAGCCGAGCGGAGCGCCCCGCGGGCCCGCCCGCGTCTGCGGCCGTTCGGGTCCTCACAGTCCCCGCGCCCGGAGTTCGC
AGCGCGCTCCAGACAAGATGGCGCGGCCGGTCCGGGGAGGGCTCGGGGCCCCGCGCCGCTCGCCTTGCCTTCTCCTTCTCTGGCT
GCTTTTGCTTCGGCTGGAGCCGGTGACCGCCGCGGCCGGCCCGCGGGCGCCCTGCGCGGCCGCCTGCACTTGCGCTGGGGACTCG
CTGGACTGCGGTGGGCGCGGGCTGGCTGCGTTGCCCGGGGACCTGCCCTCCTGGACGCGGAGCCTAAACCTGAGTTACAACAAAC
TCTCTGAGATTGACCCTGCTGGTTTTGAGGACTTGCCGAACCTACAGGAAGTGTACCTCAATAATAATGAGTTGACAGCGGTACC
ATCCCTGGGCGCTGCTTCATCACATGTCGTCTCTCTCTTTCTGCAGCACAACAAGATTCGCAGCGTGGAGGGGAGCCAGCTGAAG
GCCTACCTTTCCTTAGAAGTGTTAGATCTGAGTTTGAACAACATCACGGAAGTGCGGAACACCTGCTTTCCACACGGACCGCCTA
TAAAGGAGCTCAACCTGGCAGGCAATCGGATTGGCACCCTGGAGTTGGGAGCATTTGATGGTCTGTCACGGTCGCTGCTAACTCT
TCGCCTGAGCAAAAACAGGATCACCCAGCTTCCTGTAAGAGCATTCAAGCTACCCAGGCTGACACAACTGGACCTCAATCGGAAC
AGGATTCGGCTGATAGAGGGCCTCACCTTCCAGGGGCTCAACAGCTTGGAGGTGCTGAAGCTTCAGCGAAACAACATCAGCAAAC
TGACAGATGGGGCCTTCTGGGGACTGTCCAAGATGCATGTGCTGCACCTGGAGTACAACAGCCTGGTAGAAGTGAACAGCGGCTC
GCTCTACGGCCTCACGGCCCTGCATCAGCTCCACCTCAGCAACAATTCCATCGCTCGCATTCACCGCAAGGGCTGGAGCTTCTGC
CAGAAGCTGCATGAGTTGGTCCTGTCCTTCAACAACCTGACACGGCTGGACGAGGAGAGCCTGGCCGAGCTGAGCAGCCTGAGTG
TCCTGCGTCTCAGCCACAATTCCATCAGCCACATTGCGGAGGGTGCCTTCAAGGGACTCAGGAGCCTGCGAGTCTTGGATCTGGA
CCATAACGAGATTTCGGGCACAATAGAGGACACGAGCGGCGCCTTCTCAGGGCTCGACAGCCTCAGCAAGCTGACTCTGTTTGGA
AACAAGATCAAGTCTGTGGCTAAGAGAGCATTCTCGGGGCTGGAAGGCCTGGAGCACCTGAACCTTGGAGGGAATGCGATCAGAT
CTGTCCAGTTTGATGCCTTTGTGAAGATGAAGAATCTTAAAGAGCTCCATATCAGCAGCGACAGCTTCCTGTGTGACTGCCAGCT
GAAGTGGCTGCCCCCGTGGCTAATTGGCAGGATGCTGCAGGCCTTTGTGACAGCCACCTGTGCCCACCCAGAATCACTGAAGGGT
CAGAGCATTTTCTCTGTGCCACCAGAGAGTTTCGTGTGCGATGACTTCCTGAAGCCACAGATCATCACCCAGCCAGAAACCACCA
TGGCTATGGTGGGCAAGGACATCCGGTTTACATGCTCAGCAGCCAGCAGCAGCAGCTCCCCCATGACCTTTGCCTGGAAGAAAGA
CAATGAAGTCCTGACCAATGCAGACATGGAGAACTTTGTCCACGTCCACGCGCAGGACGGGGAAGTGATGGAGTACACCACCATC
CTGCACCTCCGTCAGGTCACTTTCGGGCACGAGGGCCGCTACCAATGTGTCATCACCAACCACTTTGGCTCCACCTATTCACATA
AGGCCAGGCTCACCGTGAATGTGTTGCCATCATTCACCAAAACGCCCCACGACATAACCATCCGGACCACCACCATGGCCCGCCT
CGAATGTGCTGCCACAGGTCACCCAAACCCTCAGATTGCCTGGCAGAAGGATGGAGGCACGGATTTCCCCGCTGCCCGTGAGCGA
CGCATGCATGTCATGCCGGATGACGACGTGTTTTTCATCACTGATGTGAAAATAGATGACGCAGGGGTTTACAGCTGTACTGCTC
AGAACTCAGCCGGTTCTATTTCAGCTAATGCCACCCTGACTGTCCTAGAGACCCCATCCTTGGTGGTCCCCTTGGAAGACCGTGT
GGTATCTGTGGGAGAAACAGTGGCCCTCCAATGCAAAGCCACGGGGAACCCTCCGCCCCGCATCACCTGGTTCAAGGGGGACCGC
CCGCTGAGCCTCACTGAGCGGCACCACTTGACCCCTGACAACCAGCTCCTGGTGGTTCAGAACGTGGTGGCAGAGGATGCGGGCC
GATATACCTGTGAGATGTCCAACACCCTGGGCACGGAGCGAGCTCACAGCCAGCTGAGCGTCCTGCCCGCAGCAGGCTGCAGGAA
GGATGGGACCACGGTAGGCATCTTCACCATTGCTGTCGTGAGCAGCATCGTCCTGACGTCACTGGTCTGGGTGTGCATCATCTAC
CAGACCAGGAAGAAGAGTGAAGAGTACAGTGTCACCAACACAGATGAAACCGTCGTGCCACCAGATGTTCCAAGCTACCTCTCTT
CTCAGGGGACCCTTTCTGACCGACAAGAAACCGTGGTCAGGACCGAGGGTGGCCCTCAGGCCAATGGGCACATTGAGAGCAATGG
TGTGTGTCCAAGAGATGCAAGCCACTTTCCAGAGCCCGACACTCACAGCGTTGCCTGCAGGCAGCCAAAGCTCTGTGCTGGGTCT
GCGTATCACAAAGAGCCGTGGAAAGCGATGGAGAAAGCTGAAGGGACACCTGGGCCACATAAGATGGAACACGGTGGCCGGGTCG
TATGCAGTGACTGCAACACCGAAGTGGACTGTTACTCCAGGGGACAAGCCTTCCACCCCCAGCCTGTGTCCAGAGACAGCGCACA
GCCAAGTGCGCCAAATGGCCCGGAGCCGGGTGGGAGTGACCAAGAGCATTCTCCACATCACCAGTGCAGCAGGACTGCCGCTGGG
TCCTGCCCCGAGTGCCAAGGGTCGCTCTACCCCAGTAACCACGATAGAATGCTGACGGCTGTGAAGAAAAAGCCAATGGCATCTC
TAGATGGGAAAGGGGATTCTTCCTGGACTTTAGCAAGGTTGTATCACCCGGACTCCACAGAGCTACAGCCTGCATCTTCATTAAC
TTCAGGCAGTCCAGAGCGCGCGGAAGCCCAGTACTTGCTTGTTTCCAATGGCCACCTCCCCAAAGCATGTGACGCCAGTCCCGAG
TCCACGCCACTGACAGGACAGCTCCCCGGGAAACAGAGGGTGCCACTGCTGTTGGCACCAAAAAGCTAGGTTTTGTCTACCTCAG
TTCTTGTCATACCAATCTCTACGGGAAAGAGAGGTAGGAGAGGCTGCGAGGAAGCTTGGGTTCAAGCGTCACTCATCTGTACATA
GTTGTAACTCCCATGTGGAGTATCAGTCGCTCACAGGACTTGG

Suplementary Figure S3. cDNA sequence of human *LRIG1* isoform A isolated from HaCaT and A431 cells. Untranslated region in blue, and additional exon in red. Underlined regions differ from isoform B. Length: 4,038 bp.

Hoesl et al.
Fig. S3
