# Supplementary material for: The transmembrane protein LRIG1 triggers melanocytic tumor development following chemically induced skin carcinogenesis
Source: Mol Oncol. 2021 Mar 31;15(8):2140–55. doi: 10.1002/1878-0261.12945 (PMC8495683; doi:10.1002/1878-0261.12945)
Supplement: Supplementary file 4 — Fig. S4. cDNA sequence of human LRIG1 isoform B. [file MOL2-15-2140-s004.pdf]

GTTCGCAGCGCGCTCCAGACAAGATGGCGCGGCCGGTCCGGGGAGGGCTCGGGGCCCCGCGCCGCTCGCCTTGCCTTCTCCTTCT
CTGGCTGCTTTTGCTTCGGCTGGAGCCGGTGACCGCCGCGGCCGGCCCGCGGGCGCCCTGCGCGGCCGCCTGCACTTGCGCTGGG
GACTCGCTGGACTGCGGTGGGCGCGGGCTGGCTGCGTTGCCCGGGGACCTGCCCTCCTGGACGCGGAGCCTAAACCTGAGTTACA
ACAAACTCTCTGAGATTGACCCTGCTGGTTTTGAGGACTTGCCGAACCTACAGGAAGTGTACCTCAATAATAATGAGTTGACAGC
GGTACCATCCCTGGGCGCTGCTTCATCACATGTCGTCTCTCTCTTTCTGCAGCACAACAAGATTCGCAGCGTGGAGGGGAGCCAG
CTGAAGGCCTACCTTTCCTTAGAAGTGTTAGATCTGAGTTTGAACAACATCACGGAAGTGCGGAACACCTGCTTTCCACACGGAC
CGCCTATAAAGGAGCTCAACCTGGCAGGCAATCGGATTGGCACCCTGGAGTTGGGAGCATTTGATGGTCTGTCACGGTCGCTGCT
AACTCTTCGCCTGAGCAAAAACAGGATCACCCAGCTTCCTGTAAGAGCATTCAAGCTACCCAGGCTGACACAACTGGACCTCAAT
CGGAACAGGATTCGGCTGATAGAGGGCCTCACCTTCCAGGGGCTCAACAGCTTGGAGGTGCTGAAGCTTCAGCGAAACAACATCA
GCAAACTGACAGATGGGGCCTTCTGGGGACTGTCCAAGATGCATGTGCTGCACCTGGAGTACAACAGCCTGGTAGAAGTGAACAG
CGGCTCGCTCTACGGCCTCACGGCCCTGCATCAGCTCCACCTCAGCAACAATTCCATCGCTCGCATTCACCGCAAGGGCTGGAGC
TTCTGCCAGAAGCTGCATGAGTTGGTCCTGTCCTTCAACAACCTGACACGGCTGGACGAGGAGAGCCTGGCCGAGCTGAGCAGCC
TGAGTGTCCTGCGTCTCAGCCACAATTCCATCAGCCACATTGCGGAGGGTGCCTTCAAGGGACTCAGGAGCCTGCGAGTCTTGGA
TCTGGACCATAACGAGATTTCGGGCACAATAGAGGACACGAGCGGCGCCTTCTCAGGGCTCGACAGCCTCAGCAAGCT**CCTTTTA**
TTAGAGCCATCCCAGTCTGCTGGCTGCAGCTCCCCGTCCCAGCCCCATATGTCTGCTGGGGGAAGGACTCTGTTTGGAAACAAGA
TCAAGTCTGTGGCTAAGAGAGCATTCTCGGGGCTGGAAGGCCTGGAGCACCTGAACCTTGGAGGGAATGCGATCAGATCTGTCCA
GTTTGATGCCTTTGTGAAGATGAAGAATCTTAAAGAGCTCCATATCAGCAGCGACAGCTTCCTGTGTGACTGCCAGCTGAAGTGG
CTGCCCCCGTGGCTAATTGGCAGGATGCTGCAGGCCTTTGTGACAGCCACCTGTGCCCACCCAGAATCACTGAAGGGTCAGAGCA
TTTTCTCTGTGCCACCAGAGAGTTTCGTGTGCGATGACTTCCTGAAGCCACAGATCATCACCCAGCCAGAAACCACCATGGCTAT
GGTGGGCAAGGACATCCGGTTTACATGCTCAGCAGCCAGCAGCAGCAGCTCCCCCATGACCTTTGCCTGGAAGAAAGACAATGAA
GTCCTGACCAATGCAGACATGGAGAACTTTGTCCACGTCCACGCGCAGGACGGGGAAGTGATGGAGTACACCACCATCCTGCACC
TCCGTCAGGTCACTTTCGGGCACGAGGGCCGCTACCAATGTGTCATCACCAACCACTTTGGCTCCACCTATTCACATAAGGCCAG
GCTCACCGTGAATGTGTTGCCATCATTCACCAAAACGCCCCACGACATAACCATCCGGACCACCACCATGGCCCGCCTCGAATGT
GCTGCCACAGGTCACCCAAACCCTCAGATTGCCTGGCAGAAGGATGGAGGCACGGATTTCCCCGCTGCCCAGACCCCATCCTTGG
TGGTCCCCTTGGAAGACCGTGTGGTATCTGTGGGAGAAACAGTGGCCCTCCAATGCAAAGCCACGGGGAACCCTCCGCCCCGCAT
CACCTGGTTCAAGGGGGACCGCCCGCTGAGCCTCACTGAGCGGCACCACTTGACCCCTGACAACCAGCTCCTGGTGGTTCAGAAC
GTGGTGGCAGAGGATGCGGGCCGATATACCTGTGAGATGTCCAACACCCTGGGCACGGAGCGAGCTCACAGCCAGCTGAGCGTCC
TGCCCGCAGCAGGCTGCAGGAAGGATGGGACCACGGTAGGCATCTTCACCATTGCTGTCGTGAGCAGCATCGTCCTGACGTCACT
GGTCTGGGTGTGCATCATCTACCAGACCAGGAAGAAGAGTGAAGAGTACAGTGTCACCAACACAGATGAAACCGTCGTGCCACCA
GATGTTCCAAGCTACCTCTCTTCTCAGGGGACCCTTTCTGACCGACAAGAAACCGTGGTCAGGACCGAGGGTGGCCCTCAGGCCA
ATGGGCACATTGAGAGCAATGGTGTGTGTCCAAGAGATGCAAGCCACTTTCCAGAGCCCGACACTCACAGCGTTGCCTGCAGGCA
GCCAAAGCTCTGTGCTGGGTCTGCGTATCACAAAGAGCCGTGGAAAGCGATGGAGAAAGCTGAAGGGACACCTGGGCCACATAAG
ATGGAACACGGTGGCCGGGTCGTATGCAGTGACTGCAACACCGAAGTGGACTGTTACTCCAGGGGACAAGCCTTCCACCCCCAGC
CTGTGTCCAGAGACAGCGCACAGCCAAGTGCGCCAAATGGCCCGGAGCCGGGTGGGAGTGACCAAGAGCATTCTCCACATCACCA
GTGCAGCAGGACTGCCGCTGGGTCCTGCCCCGAGTGCCAAGGGTCGCTCTACCCCAGTAACCACGATAGAATGCTGACGGCTGTG
AAGAAAAAGCCAATGGCATCTCTAGATGGGAAAGGGGATTCTTCCTGGACTTTAGCAAGGTTGTATCACCCGGACTCCACAGAGC
TACAGCCTGCATCTTCATTAACTTCAGGCAGTCCAGAGCGCGCGGAAGCCCAGTACTTGCTTGTTTCCAATGGCCACCTCCCCAA
AGCATGTGACGCCAGTCCCGAGTCCACGCCACTGACAGGACAGCTCCCCGGGAAACAGAGGGTGCCACTGCTGTTGGCACCAAAA
AGCTAG**GTTTTGTCTACCTCAGTTCTTGTCATACCAATCTCTACGGGAAAGAGAGGTAGGAGAGGCTGCGAGGAAGCTTGGGTTC**
AAGCGTCACTCATCTGTACATAGTTGTAACTCCCATGTGGAGTATCAGTCGCTCACAGGACTTGG

Suplementary Figure S4. cDNA sequence of human *LRIG1* isoform B isolated from A375, SK-MEL2, and SK-MEL28 cells. Untranslated region in blue, and additional exon in red. Underlined regions differ from isoform B. Length: 3,380 bp.

Hoesl et al.
Fig. S4
