# Supplementary material for: The transmembrane protein LRIG1 triggers melanocytic tumor development following chemically induced skin carcinogenesis
Source: Mol Oncol. 2021 Mar 31;15(8):2140–55. doi: 10.1002/1878-0261.12945 (PMC8495683; doi:10.1002/1878-0261.12945)
Supplement: Supplementary file 5 — Fig. S5. Amino acid sequence comparison of human LRIG1 isoforms. [file MOL2-15-2140-s002.pdf]

```
1       MARPVRGGLGAPRRSPCLLLLWLLLLRLEPVTAAAGPRAPCAAACTCAGDSLDCGGRGLA  60
        MARPVRGGLGAPRRSPCLLLLWLLLLRLEPVTAAAGPRAPCAAACTCAGDSLDCGGRGLA
1       MARPVRGGLGAPRRSPCLLLLWLLLLRLEPVTAAAGPRAPCAAACTCAGDSLDCGGRGLA  60
61      ALPGDLPSWTRSLNLSYNKLSEIDPAGFEDLPNLQEVYLNNNELTAVPSLGAASSHVVSL  120
        ALPGDLPSWTRSLNLSYNKLSEIDPAGFEDLPNLQEVYLNNNELTAVPSLGAASSHVVSL
61      ALPGDLPSWTRSLNLSYNKLSEIDPAGFEDLPNLQEVYLNNNELTAVPSLGAASSHVVSL  120
121     FLQHNKIRSVEGSQLKAYLSLEVLDLSLNNITEVRNTCFPHGPPIKELNLAGNRIGTLEL  180
        FLQHNKIRSVEGSQLKAYLSLEVLDLSLNNITEVRNTCFPHGPPIKELNLAGNRIGTLEL
121     FLQHNKIRSVEGSQLKAYLSLEVLDLSLNNITEVRNTCFPHGPPIKELNLAGNRIGTLEL  180
181     GAFDGLSRSLLTLRLSKNRITQLPVRAFKLPRLTQLDLNRNRIRLIEGLTFQGLNSLEVL  240
        GAFDGLSRSLLTLRLSKNRITQLPVRAFKLPRLTQLDLNRNRIRLIEGLTFQGLNSLEVL
181     GAFDGLSRSLLTLRLSKNRITQLPVRAFKLPRLTQLDLNRNRIRLIEGLTFQGLNSLEVL  240
241     KLQRNNISKLTDGAFWGLSKMHVLHLEYNSLVEVNSGSLYGLTALHQLHLSNNSIARIHR  300
        KLQRNNISKLTDGAFWGLSKMHVLHLEYNSLVEVNSGSLYGLTALHQLHLSNNSIARIHR
241     KLQRNNISKLTDGAFWGLSKMHVLHLEYNSLVEVNSGSLYGLTALHQLHLSNNSIARIHR  300
301     KGWSFCQKLHELVLSFNNLTRLDEESLAELSSLSVLRLSHNSISHIAEGAFKGLRSLRVL  360
        KGWSFCQKLHELVLSFNNLTRLDEESLAELSSLSVLRLSHNSISHIAEGAFKGLRSLRVL
301     KGWSFCQKLHELVLSFNNLTRLDEESLAELSSLSVLRLSHNSISHIAEGAFKGLRSLRVL  360
361     DLDHNEISGTIEDTSGAFSGLDSLSKL------------------------TLFGNKIKS  396
        DLDHNEISGTIEDTSGAFSGLDSLSKL                        TLFGNKIKS
361     DLDHNEISGTIEDTSGAFSGLDSLSKLLLLEPSQSAGCSSPSQPHMSAGGRTLFGNKIKS  420
397     VAKRAFSGLEGLEHLNLGGNAIRSVQFDAFVKMKNLKELHISSDSFLCDCQLKWLPPWLI  456
        VAKRAFSGLEGLEHLNLGGNAIRSVQFDAFVKMKNLKELHISSDSFLCDCQLKWLPPWLI
421     VAKRAFSGLEGLEHLNLGGNAIRSVQFDAFVKMKNLKELHISSDSFLCDCQLKWLPPWLI  480
457     GRMLQAFVTATCAHPESLKGQSIFSVPPESFVCDDFLKPQIITQPETTMAMVGKDIRFTC  516
        GRMLQAFVTATCAHPESLKGQSIFSVPPESFVCDDFLKPQIITQPETTMAMVGKDIRFTC
481     GRMLQAFVTATCAHPESLKGQSIFSVPPESFVCDDFLKPQIITQPETTMAMVGKDIRFTC  540
517     SAASSSSSPMTFAWKKDNEVLTNADMENFVHVHAQDGEVMEYTTILHLRQVTFGHEGRYQ  576
        SAASSSSSPMTFAWKKDNEVLTNADMENFVHVHAQDGEVMEYTTILHLRQVTFGHEGRYQ
541     SAASSSSSPMTFAWKKDNEVLTNADMENFVHVHAQDGEVMEYTTILHLRQVTFGHEGRYQ  600
577     CVITNHFGSTYSHKARLTVNVLPSFTKTPHDITIRTTTMARLECAATGHPNPQIAWQKDG  636
        CVITNHFGSTYSHKARLTVNVLPSFTKTPHDITIRTTTMARLECAATGHPNPQIAWQKDG
601     CVITNHFGSTYSHKARLTVNVLPSFTKTPHDITIRTTTMARLECAATGHPNPQIAWQKDG  660
637     GTDFPAARERRMHVMPDDDVFFITDVKIDDAGVYSCTAQNSAGSISANATLTVLETPSLV  696
        GTDFPAA                                              +TPSLV
661     GTDFPAA-----------------------------------------------QTPSLV  673
697     VPLEDRVVSVGETVALQCKATGNPPPRITWFKGDRPLSLTERHHLTPDNQLLVVQNVVAE  756
        VPLEDRVVSVGETVALQCKATGNPPPRITWFKGDRPLSLTERHHLTPDNQLLVVQNVVAE
674     VPLEDRVVSVGETVALQCKATGNPPPRITWFKGDRPLSLTERHHLTPDNQLLVVQNVVAE  733
757     DAGRYTCEMSNTLGTERAHSQLSVLPAAGCRKDGTTVGIFTIAVVSSIVLTSLVWVCIIY  816
        DAGRYTCEMSNTLGTERAHSQLSVLPAAGCRKDGTTVGIFTIAVVSSIVLTSLVWVCIIY
734     DAGRYTCEMSNTLGTERAHSQLSVLPAAGCRKDGTTVGIFTIAVVSSIVLTSLVWVCIIY  793
817     QTRKKSEEYSVTNTDETVVPPDVPSYLSSQGTLSDRQETVVRTEGGPQANGHIESNGVCP  876
        QTRKKSEEYSVTNTDETVVPPDVPSYLSSQGTLSDRQETVVRTEGGPQANGHIESNGVCP
794     QTRKKSEEYSVTNTDETVVPPDVPSYLSSQGTLSDRQETVVRTEGGPQANGHIESNGVCP  853
877     RDASHFPEPDTHSVACRQPKLCAGSAYHKEPWKAMEKAEGTPGPHKMEHGGRVVCSDCNT  936
        RDASHFPEPDTHSVACRQPKLCAGSAYHKEPWKAMEKAEGTPGPHKMEHGGRVVCSDCNT
854     RDASHFPEPDTHSVACRQPKLCAGSAYHKEPWKAMEKAEGTPGPHKMEHGGRVVCSDCNT  913
937     EVDCYSRGQAFHPQPVSRDSAQPSAPNGPEPGGSDQEHSPHHQCSRTAAGSCPECQGSLY  996
        EVDCYSRGQAFHPQPVSRDSAQPSAPNGPEPGGSDQEHSPHHQCSRTAAGSCPECQGSLY
914     EVDCYSRGQAFHPQPVSRDSAQPSAPNGPEPGGSDQEHSPHHQCSRTAAGSCPECQGSLY  973
997     PSNHDRMLTAVKKKPMASLDGKGDSSWTLARLYHPDSTELQPASSLTSGSPERAEAQYLL  1056
        PSNHDRMLTAVKKKPMASLDGKGDSSWTLARLYHPDSTELQPASSLTSGSPERAEAQYLL
974     PSNHDRMLTAVKKKPMASLDGKGDSSWTLARLYHPDSTELQPASSLTSGSPERAEAQYLL  1033
1057    VSNGHLPKACDASPESTPLTGQLPGKQRVPLLLAPKS  1093
        VSNGHLPKACDASPESTPLTGQLPGKQRVPLLLAPKS
1034    VSNGHLPKACDASPESTPLTGQLPGKQRVPLLLAPKS  1070
```

Suplementary Figure S5. Amino acid sequence comparison of human LRIG1 isoform A (blue) and isoform B (green).

Hoesl et al.
Fig. S5
