# Supplementary material for: The transmembrane protein LRIG1 triggers melanocytic tumor development following chemically induced skin carcinogenesis
Source: Mol Oncol. 2021 Mar 31;15(8):2140–55. doi: 10.1002/1878-0261.12945 (PMC8495683; doi:10.1002/1878-0261.12945)
Supplement: Supplementary file 6 — Fig. S6. Generation of A375 LRIG1 knockout cell lines. [file MOL2-15-2140-s010.pdf]

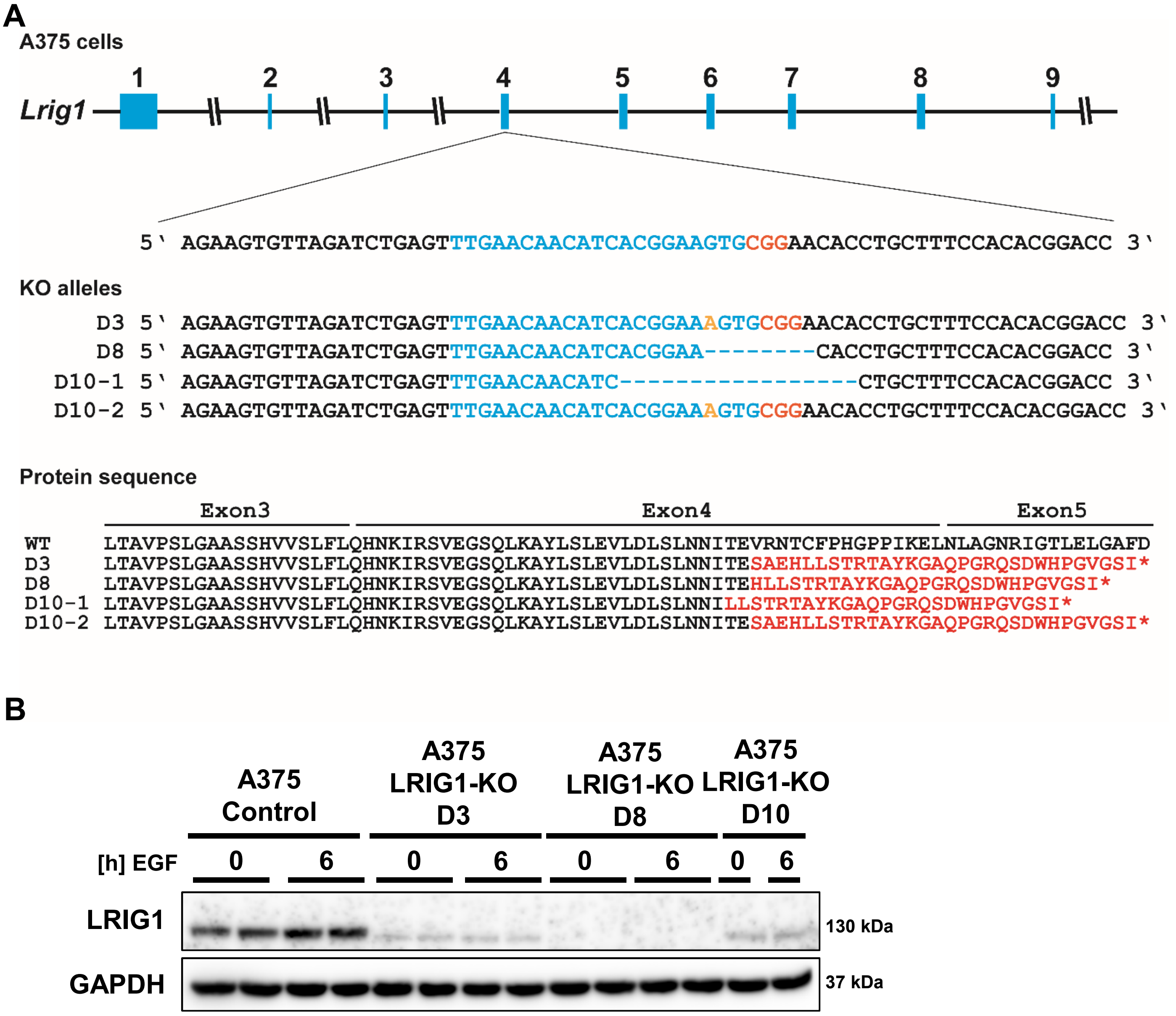

Supplementary Figure S6: (A) Generation of A375 LRIG1 knockout cell lines using CRISPR/Cas9 technology. Partial DNA sequence of *LRIG1* exon 4. The sgRNA binding site is indicated in blue, the protospacer adjacent motif (PAM) in red. Insertions (orange) or deletions (-) lead a shift of reading frame. Partial amino acid sequences encoded by the wild type (WT) and mutant *LRIG1* alleles. WT LRIG1 aa sequence in black, missense aa sequence in red, and the premature termination codon as asterisk. (B) LRIG1 deletion in three different A375 clones unstimulated and 6 h after EGF stimulation. Western blot analysis of LRIG1 protein. GAPDH was used as reference protein.

Hoesl et al.
Fig. S6
