# Supplementary material for: The transmembrane protein LRIG1 triggers melanocytic tumor development following chemically induced skin carcinogenesis
Source: Mol Oncol. 2021 Mar 31;15(8):2140–55. doi: 10.1002/1878-0261.12945 (PMC8495683; doi:10.1002/1878-0261.12945)
Supplement: Supplementary file 7 — Fig. S7. Western blots of HaCaT and A375 cell lines. [file MOL2-15-2140-s003.pdf]

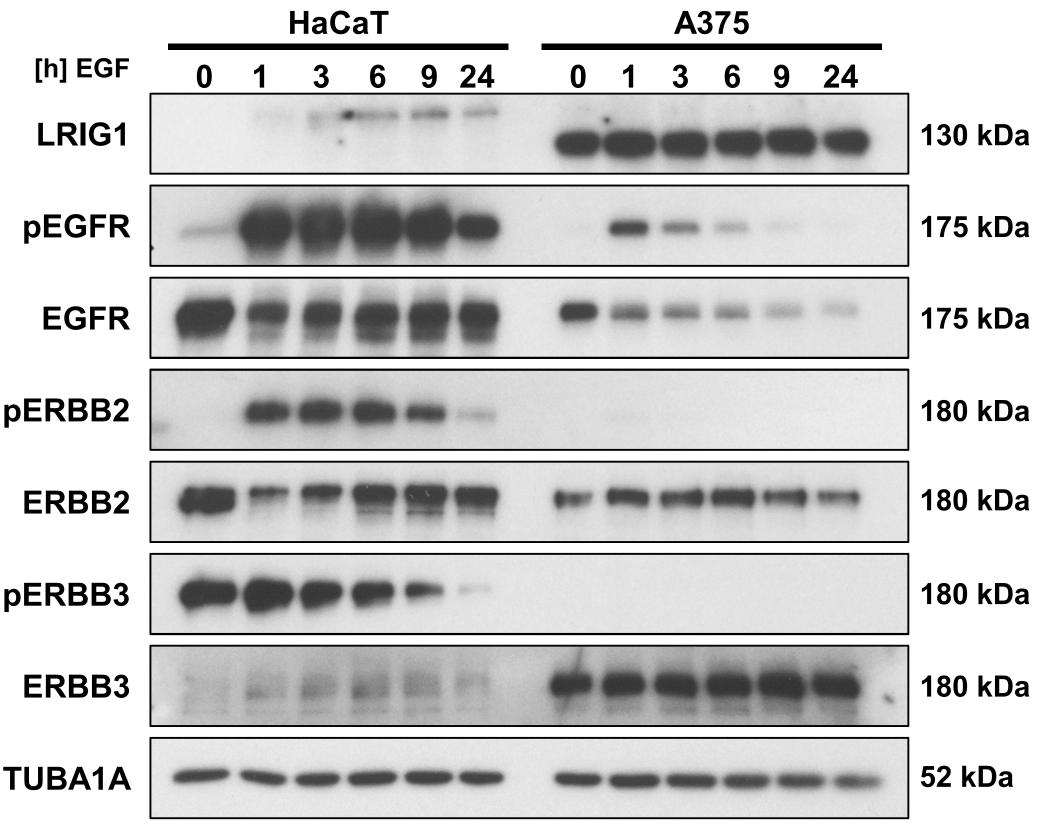

Suplementary Figure S7. LRIG1 expression is consistent high in A375 melanoma cells without and after EGF stimulation, but increases in human keratinocytes (HaCaT) six hours after EGF stimulation. Western blot analysis of LRIG1 protein and ERBB receptors in HaCaT and A375 cells 0h, 1h, 3h, 6h, 9h and 24h after EGF stimulation. TUBA1A was used as reference protein.

Hoesl et al.
Fig. S7
