# Supplementary material for: The transmembrane protein LRIG1 triggers melanocytic tumor development following chemically induced skin carcinogenesis
Source: Mol Oncol. 2021 Mar 31;15(8):2140–55. doi: 10.1002/1878-0261.12945 (PMC8495683; doi:10.1002/1878-0261.12945)
Supplement: Supplementary file 8 — Fig. S8. Immunohistochemical labeling of LRIG1 in human cSCC and melanoma. [file MOL2-15-2140-s007.pdf]

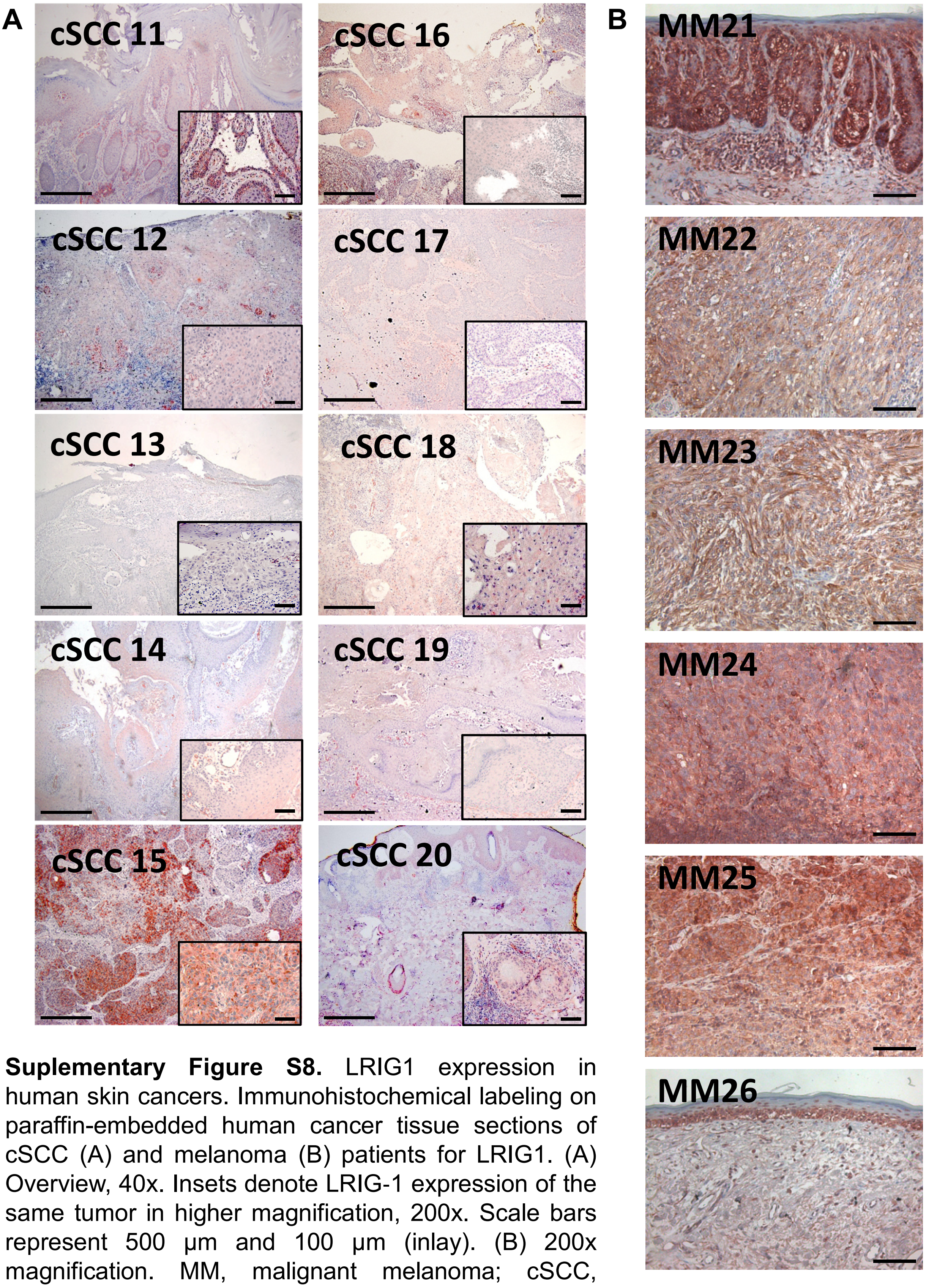

Suplementary Figure S8. LRIG1 expression in human skin cancers. Immunohistochemical labeling on paraffin-embedded human cancer tissue sections of cSCC (A) and melanoma (B) patients for LRIG1. (A) Overview, 40x. Insets denote LRIG-1 expression of the same tumor in higher magnification, 200x. Scale bars represent 500 µm and 100 µm (inlay). (B) 200x magnification. MM, malignant melanoma; cSCC, cutaneous squamous cell carcinoma. Scale bars represent 100 µm.

Hoesl et al.
Fig. S8
