# Supplementary material for: The transmembrane protein LRIG1 triggers melanocytic tumor development following chemically induced skin carcinogenesis
Source: Mol Oncol. 2021 Mar 31;15(8):2140–55. doi: 10.1002/1878-0261.12945 (PMC8495683; doi:10.1002/1878-0261.12945)
Supplement: Supplementary file 9 — Table S1. Antibodies employed for Western blots analysis, immunoprecipitation, immunohistochemistry, and immunofluorescence. [file MOL2-15-2140-s009.docx]

**Table S1.** Antibodies employed for Western blots analysis, immunoprecipitation, immunohistochemistry, and immunofluorescence.

| **Antigen** | **Antibody** | **App.** | **Host** | **Dilution** |
| --- | --- | --- | --- | --- |
| LRIG1 | R&D Systems, Minneapolis, MN, USA, #AF3688 | WB | goat | 1:2500 |
| LRIG1 | Cell Signaling, Boston, MA, USA, #12752 | WB | rabbit | 1:1000 |
| LRIG1 | Santa Cruz, Heidelberg, Germany, #134435 | IF | rabbit | 1:200 |
| LRIG1 | Novus Biologicals, Centennial, CO, USA, #NBP2‑30926 | IHC | rabbit | 1:300 |
| p-EGFR (Tyr 1068) | Cell Signaling, Boston, MA, USA, #3777 | WB | rabbit | 1:1000 |
| EGFR | Santa Cruz, Heidelberg, Germany, #03, | WB | rabbit | 1:500 |
| EGFR | Cell Signaling, Boston, MA, USA, #2232 | WB | rabbit | 1:1000 |
| p-ERBB2 (Tyr 877) | Cell Signaling, Boston, MA, USA, #2241 | WB | rabbit | 1:1000 |
| ERBB2 | Cell Signaling, Boston, MA, USA, #4290 | WB | rabbit | 1:1000 |
| p-ERBB3 (Tyr 1289) | Cell Signaling, Boston, MA, USA, #4791 | WB | rabbit | 1:1000 |
| ERBB3 | Cell Signaling, Boston, MA, USA, #12708 | WB | rabbit | 1:1000 |
| NOTCH1 | Cell Signaling, Boston, MA, USA, #3608 | WB | rabbit | 1:1000 |
| p-MAPK1/2 (Thr202/Tyr204) | Cell Signaling, Boston, MA, USA, #4370 | WB | rabbit | 1:1000 |
| MAPK1/2 | Cell Signaling, Boston, MA, USA, #9102 | WB | rabbit | 1:2000 |
| p-MAPK14(p-p38 MAPK) | Cell Signaling, Boston, MA, USA, #9211 | WB | rabbit | 1:1000 |
| MAPK14 (p38 MAPK) | Cell Signaling, Boston, MA, USA, #9212 | WB | rabbit | 1:1000 |
| p-PTEN (Ser380/Thr382/383) | Cell Signaling, Boston, MA, USA, #9554 | WB | rabbit | 1:1000 |
| PTEN | Cell Signaling, Boston, MA, USA, #9552 | WB | rabbit | 1:1000 |
| p-P53 | Cell Signaling, Boston, MA, USA, #12571 | WB | rabbit | 1:1000 |
| P53 | Cell Signaling, Boston, MA, USA, #2524 | WB | mouse | 1:1000 |
| CASP3 | Cell Signaling, Boston, MA, USA, #9662 | WB | rabbit | 1:1000 |
| MLANA | Abcam, Cambridge, UK, #ab210546 | WB/IF | rabbit | WB: 1:2000  IF: 1:1000 |
| MKI67 | Dianova, Hamburg, Germany, #M7249 | IHC | rat | 1:200 |
| KRT5 | BioLegend, San Diego, CA, USA, #905501 | IHC | rabbit | 1:800 |
| KRT6 | BioLegend, San Diego, CA, USA, #905701 | IHC | rabbit | 1:800 |
| KRT10 | BioLegend, San Diego, CA, USA, #905701 | IHC | rabbit | 1:800 |
| LOR | BioLegend, San Diego, CA, USA, #905101 | IHC | rabbit | 1:800 |
| TUBA1A | Cell Signaling, Boston, MA, USA, #2125 | WB | rabbit | 1:1000 |
| GAPDH | Cell Signaling, Boston, MA, USA, #2118 | WB | rabbit | 1:5000 |
| donkey α rabbit | Jackson ImmunoResearch, Ely, UK‚ #711-546-152 | IF | donkey | 1:1000 |
| mouse α rat | Jackson ImmunoResearch, Ely, UK‚ #212-066-168 | IHC | mouse | 1:100 |
| goat α rabbit ( SignalStain® Boost IHC Detection Reagent) | Cell Signaling, Boston, MA, USA, #8114 | IHC | goat | RTU |
| rabbit α mouse | Cell Signaling, Boston, MA, USA, #7076 | WB | rabbit | 1:2500 |
| goat α rabbit | Cell Signaling, Boston, MA, USA, #7074 | WB | goat | 1:2500 |
| donkey α goat | R&D Systems, Minneapolis, MN, USA, #HAF109 | WB | donkey | 1:2500 |
