# Supplementary material for: The transmembrane protein LRIG1 triggers melanocytic tumor development following chemically induced skin carcinogenesis
Source: Mol Oncol. 2021 Mar 31;15(8):2140–55. doi: 10.1002/1878-0261.12945 (PMC8495683; doi:10.1002/1878-0261.12945)
Supplement: Supplementary file 10 — Table S2. Total spectrum counts of LRIG1 peptides. [file MOL2-15-2140-s006.docx]

**Table S2.** Total spectrum counts of LRIG1 peptides.

LRIG1-TG TPA treated 130 kDA

Total spectrum count: 13

| **Sequence** | **Prob** | **Mascot Ion score** | **Observed** | **Actual Mass** | **Charge** | **Delta Da** | **Delta PPM** | **Start** | **Stop** |
| --- | --- | --- | --- | --- | --- | --- | --- | --- | --- |
| (K)ILSVDGSQLK(S) | 100% | 27,67 | 530,304 | 1.058,59 | 2 | -0,003938 | -3,716 | 129 | 138 |
| (R)IRELNLASNR(I) | 98% | 18,16 | 395,8946 | 1.184,66 | 3 | -0,0006729 | -0,5676 | 167 | 176 |
| (R)ISILESGAFDGLSR(S) | 100% | 31,14 | 732,8882 | 1.463,76 | 2 | -0,0004159 | -0,284 | 177 | 190 |
| (R)LTQLDLNR(N) | 99% | 34,68 | 486,7772 | 971,5399 | 2 | -0,0003059 | -0,3146 | 215 | 222 |
| (R)LTDGAFWGLSK(M) | 100% | 43,13 | 597,8113 | 1.193,61 | 2 | -0,0003159 | -0,2645 | 252 | 262 |
| (R)LSHNAISHIAEGAFK(G) | 98% | 23,47 | 532,2838 | 1.593,83 | 3 | 0,003241 | 2,032 | 340 | 354 |
| (K)DNEVLANADmENFAHVR(A) | 100% | 59,61 | 654,2986 | 1.959,87 | 3 | -0,000561 | -0,2861 | 535 | 551 |
| (R)AQDGEVmEYTTILHLR(H) | 100% | 28,85 | 631,3113 | 1.890,91 | 3 | -0,002593 | -1,371 | 552 | 567 |
| (R)LTVNVLPSFTK(I) | 99% | 22,55 | 609,858 | 1.217,70 | 2 | -0,0006059 | -0,4972 | 595 | 605 |
| (K)IPHDIAIR(T) | 99% | 32,44 | 467,7769 | 933,5393 | 2 | -0,0004579 | -0,49 | 606 | 613 |
| (R)DSGQPGTASSQELR(Q) | 100% | 37,21 | 716,8366 | 1.431,66 | 2 | -0,0006039 | -0,4216 | 948 | 961 |
| (R)RGPLLLAPR(-) | 97% | 17,97 | 496,8213 | 991,628 | 2 | -0,001232 | -1,241 | 1082 | 1090 |
| (R)GPLLLAPR(-) | 99% | 39,38 | 418,7709 | 835,5272 | 2 | -0,0009439 | -1,128 | 1083 | 1090 |

LRIG1-TG TPA treated 100 kDA

Total spectrum count: 12

| **Sequence** | **Prob** | **Mascot Ion score** | **Observed** | **Actual Mass** | **Charge** | **Delta Da** | **Delta PPM** | **Start** | **Stop** |
| --- | --- | --- | --- | --- | --- | --- | --- | --- | --- |
| (K)ILSVDGSQLK(S) | 100% | 31,94 | 530,3059 | 1.058,60 | 2 | -0,0001059 | -0,09998 | 129 | 138 |
| (R)IRELNLASNR(I) | 100% | 34,07 | 395,8948 | 1.184,66 | 3 | 0,00007106 | 0,05993 | 167 | 176 |
| (R)ISILESGAFDGLSR(S) | 100% | 47,33 | 732,888 | 1.463,76 | 2 | -0,0006999 | -0,4779 | 177 | 190 |
| (R)LTQLDLNR(N) | 99% | 47,54 | 486,7769 | 971,5392 | 2 | -0,0009459 | -0,9726 | 215 | 222 |
| (R)LDEESLAELSSLSILR(L) | 100% | 32,41 | 887,9755 | 1.773,94 | 2 | 0,0002061 | 0,1161 | 324 | 339 |
| (R)LSHNAISHIAEGAFK(G) | 97% | 18,79 | 532,283 | 1.593,83 | 3 | 0,0006911 | 0,4333 | 340 | 354 |
| (R)VLDLDHNEISGTIEDTSGAFTGLDNLSK(L) | 97% | 17,9 | 987,8165 | 2.960,43 | 3 | 0,002039 | 0,6885 | 361 | 388 |
| (R)AFSGLESLEHLNLGENAIR(S) | 99% | 23,62 | 1.035,53 | 2.069,05 | 2 | -0,0001999 | -0,09659 | 403 | 421 |
| (R)SVQFDAFAK(M) | 100% | 24,16 | 506,7591 | 1.011,50 | 2 | 0,001024 | 1,011 | 422 | 430 |
| (K)DNEVLANADmENFAHVR(A) | 100% | 27,58 | 654,2987 | 1.959,87 | 3 | -0,000195 | -0,09942 | 535 | 551 |
| (R)AQDGEVmEYTTILHLR(H) | 100% | 55,27 | 631,3118 | 1.890,91 | 3 | -0,001126 | -0,5951 | 552 | 567 |
| (K)IPHDIAIR(T) | 99% | 32,16 | 467,7768 | 933,539 | 2 | -0,0007239 | -0,7746 | 606 | 613 |

LRIG1-TG untreated 130 kDA

Total spectrum count: 5

| **Sequence** | **Prob** | **Mascot Ion score** | **Observed** | **Actual Mass** | **Charge** | **Delta Da** | **Delta PPM** | **Start** | **Stop** |
| --- | --- | --- | --- | --- | --- | --- | --- | --- | --- |
| (R)ISILESGAFDGLSR(S) | 100% | 31,06 | 732,8879 | 1.463,76 | 2 | -0,0009359 | -0,639 | 177 | 190 |
| (R)LTQLDLNR(N) | 99% | 41,5 | 486,7768 | 971,5391 | 2 | -0,001064 | -1,094 | 215 | 222 |
| (R)LTDGAFWGLSK(M) | 100% | 23,84 | 597,8113 | 1.193,61 | 2 | -0,0003019 | -0,2528 | 252 | 262 |
| (R)VLDLDHNEISGTIEDTSGAFTGLDNLSK(L) | 95% | 16,53 | 987,8162 | 2.960,43 | 3 | 0,001346 | 0,4545 | 361 | 388 |
| (R)GPLLLAPR(-) | 99% | 46,06 | 418,771 | 835,5274 | 2 | -0,0007639 | -0,9132 | 1083 | 1090 |

LRIG1-TG untreated 100 kDA

Total spectrum count: 1

| **Sequence** | **Prob** | **Mascot Ion score** | **Observed** | **Actual Mass** | **Charge** | **Delta Da** | **Delta PPM** | **Start** | **Stop** |
| --- | --- | --- | --- | --- | --- | --- | --- | --- | --- |
| (R)LTQLDLNR(N) | 99% | 32,19 | 486,7772 | 971,5398 | 2 | -0,0004139 | -0,4256 | 215 | 222 |
